# A novel RIP1-mediated canonical WNT signaling pathway that promotes colorectal cancer metastasis via β -catenin stabilization-induced EMT

**DOI:** 10.1038/s41417-023-00647-6

**Published:** 2023-07-27

**Authors:** A-Ram Kang, Jung-Lim Kim, YoungHa Kim, Sanghee Kang, Sang-Cheul Oh, Jong Kuk Park

**Affiliations:** 1 Division of Radiation Biomedical Research, Korea Institute of Radiological and Medical Sciences, Seoul, Republic of Korea; 2 Division of Oncology/Hematology, Department of Internal Medicine, College of Medicine, Korea University, Seoul, Republic of Korea; 3 Division of Colon and Rectal Surgery, Department of Surgery, Guro Hospital, Korea University College of Medicine, Korea University, Seoul, Republic of Korea

**Keywords:** Cancer microenvironment, Cancer microenvironment

## Abstract

RIP1 (receptor-interacting protein kinase 1) is an important component of TNF-α signaling that contributes to various pathological effects. Here, we revealed new potential roles of RIP1 in controlling WNT/β-catenin canonical signaling to enhance metastasis of colorectal cancer (CRC). First, we showed that WNT3A treatment sequentially increased the expression of RIP1 and β-catenin. Immunohistochemical analyses of human CRC tissue arrays consisting of normal, primary, and metastatic cancers indicated that elevated RIP1 expression might be related to β-catenin expression, carcinogenesis, and metastasis. Intravenous injection of RIP1 over-expressed CRC cells into mice has demonstrated that RIP1 may promote metastasis. Immunoprecipitation (IP) results indicated that WNT3A treatment induces direct binding between RIP1 and β-catenin, and that this stabilizes the β-catenin protein in a manner that depends on the regulation of RIP1 ubiquitination via downregulation of the E3 ligase, cIAP1/2. Elimination of cIAP1/2 expression and inhibition of its ubiquitinase activity enhance WNT3A-induced RIP1 and β-catenin protein expression and binding, which stimulates endothelial-mesenchymal transition (EMT) induction to enhance the migration and invasion of CRC cells in vitro. The results of the in vitro binding assay and IP of exogenous RIP1-containing CRC cells additionally verified the direct binding of RIP1 and β-catenin. RIP1 expression can destroy the β-catenin–β-TrCP complex. Taken together, these results suggest a novel EMT-enhancing role of RIP1 in the WNT pathway and suggest a new canonical WNT3A–RIP1–β-catenin pathway that contributes to CRC malignancy by promoting EMT.

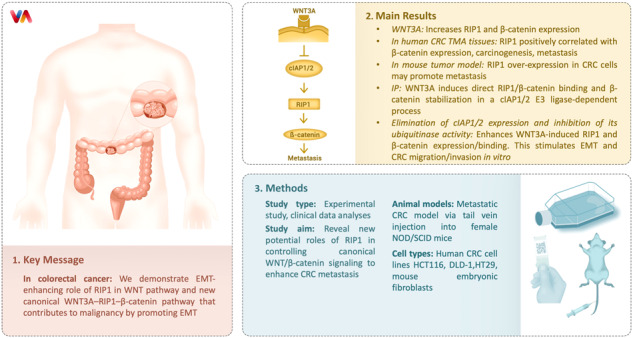

## Introduction

Colorectal cancer (CRC) is one of the most common human malignancies, and its incidence is steadily rising [1]. Despite the development of new treatment regimens, there is no effective therapy for advanced CRC with metastasis [2, 3]. Therefore, understanding the molecular mechanisms underlying the onset of metastasis in CRC, particularly how CRC cells acquire invasive and metastatic properties in their microenvironment, is important for developing optimized strategies aimed at treating CRC. The Wnt/β-catenin signaling pathway has important functions in the tumorigenesis, invasion, and metastasis of CRC. Dysregulation of the canonical Wnt/β-catenin signaling pathway can easily lead to cell proliferation, tumorigenesis, and epithelial-mesenchymal transition (EMT) induction in CRC [4–8]. Previous studies have demonstrated that inhibition of Wnt/β-catenin signaling reduces EMT and inhibits breast cancer metastasis [9].

Receptor interaction protein kinase 1 (RIP1) is an important upstream kinase of various inflammation signals, including those mediated by TLR3, TNF-α, IL-6, etc [10–12]. RIP1 is located at a critical position in these pathways; it may regulate various intracellular molecules in NF-κB- and MAPK-related cell survival signaling, while functioning as a major mediator of apoptosis- and necroptosis-inducing machineries [11–14]. Another role is also seen in the involvement of RIP1 in cancer proliferation, such as melanoma, gallbladder cancer (GBC), and ovarian cancer [15–17]. During GBC invasion, RIP1 regulates TNF-α-mediated lymphangiogenesis and lymph node metastasis by regulating the NF-κB–VEGF-C pathway [18]. We previously showed that RIP1 is a novel component of the γ-ionizing radiation-induced invasion of non-small cell lung cancer cells [19] and that radiation-induced IL-1β expression and secretion promotes cancer cell migration/invasion via activation of the NF-kB–RIP1 pathway [20]. RIP1 is overexpressed in advanced-stage lung carcinomas, but the underlying mechanism is unknown.

Based on these previous reports, we hypothesized that RIP1 contributes to CRC metastasis regulated by WNT/β-catenin signaling [21]. Here, we report that WNT3A treatment of CRC cells increases the levels of RIP1 and its binding to β-catenin, which promotes EMT and migration/invasion in vitro and in vivo. Furthermore, we revealed that WNT3A decreases cIAP1/2 expression to block RIP1 ubiquitination, thereby increasing β-catenin stability. RIP1 overexpression also decreased the expression of β-TrCP, an inhibitory binding molecule of β-catenin, and dissociated its binding to β-catenin. Our results suggest that RIP1 plays an important role in CRC metastasis by regulating WNT/β-catenin signaling, and therefore could be a potential therapeutic target for treating CRC.

## Methods and materials

### Cell culture and transfection

The human colorectal cancer cells cell lines, HCT116 and DLD-1, were purchased from the American Type Culture Collection (Rockville, MD, USA) and maintained in an incubator using RPMI-1640 medium (Corning, Manassas, VA, USA) supplemented with 10% fetal bovine serum (FBS; Gibco, Grand Island, NY, USA) at 37 °C with 5% CO_2_. Mouse embryonic fibroblasts (MEFs; wtMEF and *RIP1*-/- MEF) were obtained from Dr. Zheng-Gang Liu (NIH/NCI) and maintained using Dulbecco’s modified Eagle’s medium (DMEM; Corning, Manassas, VA, USA) supplemented with 10% FBS in an incubator at 37 °C with 5% CO_2_. Transfection was performed as described previously [22]. HT29-Luc-^Ctr^ and HT29-Luc-^*Flag-RIP1*^ stable cells were generated from HT29-Luciferase containing cells (obtained from Dr. S.G. Oh, Korea University, Seoul, Korea). The cells were transfected with a Flag-pcDNA3.1 or Flag-*RIP1* plasmids (Watertown, MA, USA) and selected using G418 selection. DLD-1^*shcon*^ and DLD-1^*shRIP1*^ were constructed from DLD-1 cells by transfection with the non-silencing pGIPZ lentiviral shRNA control vector (Addgene_121488) and pGIPZ lentiviral shRNA RIP1, respectively. After repeated selection with puromycin (ABM, Richmond, BC, Canada), IB revealed stable inhibition of RIP1 expression in the transduced cell lines, which were designated DLD-1^*shcon*^, DLD-1^*shRIP1-#1*^, DLD-1^*shRIP1-*#2^ and DLD-1^*shRIP1-#3*^ (data not shown). We used DLD-1^*shcon*^ and DLD-1^*shRIP1-#1*^ for migration and invasion assays.

### Plasmids and reagents

HA-tagged *ubiquitin* plasmids (pRK5-HA-*Ubiquitin*) were provided by Dr. Seongman Kang (Korea University). The siRNA constructs for *RIP1, cIAP1, cIAP2*, and non-targeted control were obtained from Santa Cruz Biotechnology, Inc. (Dallas, TX, USA). The non-silencing pGIPZ lentiviral shRNA control vector and GIPZ lentiviral *RIP1* shRNAs were purchased from Dharmacon (Lafayette, CO, USA). Human WNT3A (hWNT3A) was purchased from R&D Systems (Minneapolis, MN, USA). Cycloheximide (CHX) and the proteasome inhibitor MG132 were purchased from Sigma Aldrich (St. Louis, MO, USA) and EMD Millipore Corp Chemical (Houston, TX, USA), respectively. MG132 was dissolved in dimethyl sulfoxide (DMSO) as a 10mM stock solution and stored at −20 °C.

### Immunoblotting analysis

IB analyses were conducted as previously described [23]. Anti-RIP1, E-cadherin, and vimentin antibodies were purchased from BD Biosciences (San Diego, CA, USA); anti-β-catenin and β-TrCP antibodies from Cell Signaling Technology, Inc. (Beverly, MA, USA); anti-HA, ubiquitin, β-catenin, cIAP1, cIAP2, and GST antibodies from Santa Cruz Biotechnology, Inc.; anti-vimentin and HIS antibodies from Abcam (Cambridge, UK); and anti-Flag and β-actin antibodies were used as a loading control (Sigma-Aldrich).

### Immunoprecipitation analysis

Immunoprecipitation (IP) experiments were performed as described previously [22]. Briefly, HCT116 and DLD-1 cells were transiently transfected with Flag-*Mock* (*pcDNA3.1*) or Flag-*RIP1*. After 48 h, cells were harvested and lysed on ice for 60 min in 0.7% NP-40 lysis buffer supplemented with protease inhibitors (10 µg/ml aprotinin, 10 µg/ml leupeptin, and 2 mM PMSF). The resulting lysates were centrifuged at 13,000 × *g* for 20 min at 4 °C, and the supernatants were incubated with the anti- β-catenin antibody at 4 °C overnight. Protein G-Sepharose beads (GE Healthcare, Little Chalfont, UK) were then added, and bead-bound proteins were analyzed using sodium dodecyl sulfate (SDS)–polyacrylamide gel electrophoresis.

### Protein degradation assay

HCT116 and DLD-1 cells were serum-starved for 16 h and then treated with or without WNT3A (100 ng/ml) for 2 h. After 24 h, the cells were treated with 100 μg/mL cycloheximide (CHX; Sigma-Aldrich, St. Louis, MO, USA) to inhibit protein synthesis. Cycloheximide-treated cells were collected at the indicated time points (0, 12, and 24 h) and processed for IB using antibodies against RIP1, β-catenin, and β-actin.

### In vivo ubiquitylation assay

In vivo ubiquitylation assays were performed as previously described [23]. HCT116 and DLD-1 cells were transiently transfected with pRK5-HA-*Ubiquitin* for 24 h, followed by incubation with the proteasome inhibitor MG132 (10 µM) for 7 h. Cells were lysed for 60 min at 4 °C in a RIPA buffer (20 mM Tris-Cl, 150 mM NaCl, 0.1% SDS, 1% Triton X-100, 1% sodium deoxycholate, pH 7.5) containing the indicated protease inhibitor. Protein concentrations were determined using the Bio-Rad Protein Assay Kit (Bio-Rad Laboratories, Hercules, CA, USA). Cell lysates were immunoprecipitated using an anti-RIP1 antibody, after which the precipitated proteins were subjected to IB analysis and the blots were probed using an anti-HA antibody.

### In vitro binding assay

For the in vitro binding assays, 200 ng of GST-tagged β-catenin (Abnova, Taiwan) was incubated with 200 ng of HIS-tagged RIP1 (Origene, Rockville, MD, USA), in different protein combinations, in 150 μl of cold buffer A (50 mM Tris-HCl pH 7.5, 100 mM NaCl, 10% glycerol) containing 0.1% Triton X-100 for 1.5 h at 4 °C. Ni-NTA (nickel-nitrilotriacetic agarose; Qiagen, Germantown, MD, USA) was added, the mixtures were incubated with the proteins for 3 h at 4 °C, and centrifugation was performed at 1000×*g* for 5 min. The beads were then washed five times with five bead volumes of the Ni-NTA resin wash buffer. The bead-bound proteins were analyzed by IB using anti-GST and anti-HIS antibodies, according to standard protocols.

### Migration and invasion assays

Migration and invasion assays were performed as previously described [19]. WNT3A-treated or untreated DLD-1 cells (2 × 10^4^) in 200 μL serum-free medium were added to 0.1% bovine serum albumin and seeded into the upper chamber of a transwell system.

### Tissue microarrays and immunohistochemistry

Human CRC TMA (CDA3) was purchased from SUPER BIO CHIPS (Seoul, Korea) and CO952a TMA was obtained from US Biomax (Derwood, MD, USA). Immunohistochemistry (IHC) was performed as previously described [24].

### Metastatic animal model

The protocols of all animal experiments were approved by the Institutional Animal Care and Use Committee (IACUC No. Kirams 2020-0052). Female NOD/SCID mice (5–6-week old) were purchased from GemPharmatech (Shanghai, China). HT29-Luciferase-pcDNA3.1 (HT29-Luc-^*Ctr*^) or HT29-Luciferase-RIP1 (HT29-Luc-^*RIP1*^) cells stably expressing pcDNA3.1 or RIP1 were injected into 20 female NOD/SCID mice through the tail vein (1 × 10^7^ cells/mouse). Mice injected with these cells were separated into two groups (10 mice/group): control (HT29-Luc^-*Ctr*^) and RIP1 (HT29-Luc-^*RIP1*^). Tumor metastasis was assessed using an In-Vivo Xtreme bioluminescence system (Bruker, Billerica, MA, USA) starting on day 1 and at 2-week intervals for 10 weeks. d-Luciferin (150 mg/kg; GOLDBIO, St. Louis, MO, USA) was injected 15 min prior to each bioluminescence scan.

### Analysis of panels

IHC analysis of Human CRC (CDA3) and CO952a TMAs from US Biomax (Derwood, MD, USA) were performed as follows: The stained TMAs were evaluated with an Olympus BX50 light microscope (Bustleton Pike Feasterville, PA, USA). For semi-quantitative evaluation of the slides, multi-score of staining frequency and intensity was applied. The percentage of positive cells was divided into five grades (percentage scores), as follows: 0 (<10%); 1 (10–25%); 2 (26–50%); 3 (51–75%); and 4 (>75%). The staining intensity was rated as follows: 0 (no staining); 1 (light brown); 2 (brown); and 3 (dark brown). Positive staining was determined using the following formula: overall score = percentage score × intensity score. Expression was classified into two groups (positive and negative) with a cut-off value based on the median value of the respective overall score.

### Quantification and statistical analysis

Data were analyzed using the GraphPad Prism software (La Jolla, CA, USA; GraphPad Prism). The significance of differences between experimental groups was determined using Student’s *t*-test. Error bars indicate standard deviations (SD). Individual *p*-values are denoted by asterisks (*p < 0.05, **p < 0.01, and ***p < 0.001).

## Results

### RIP1 could be involved in WNT/β-catenin signaling in colorectal cancer

**Fig. 1:**
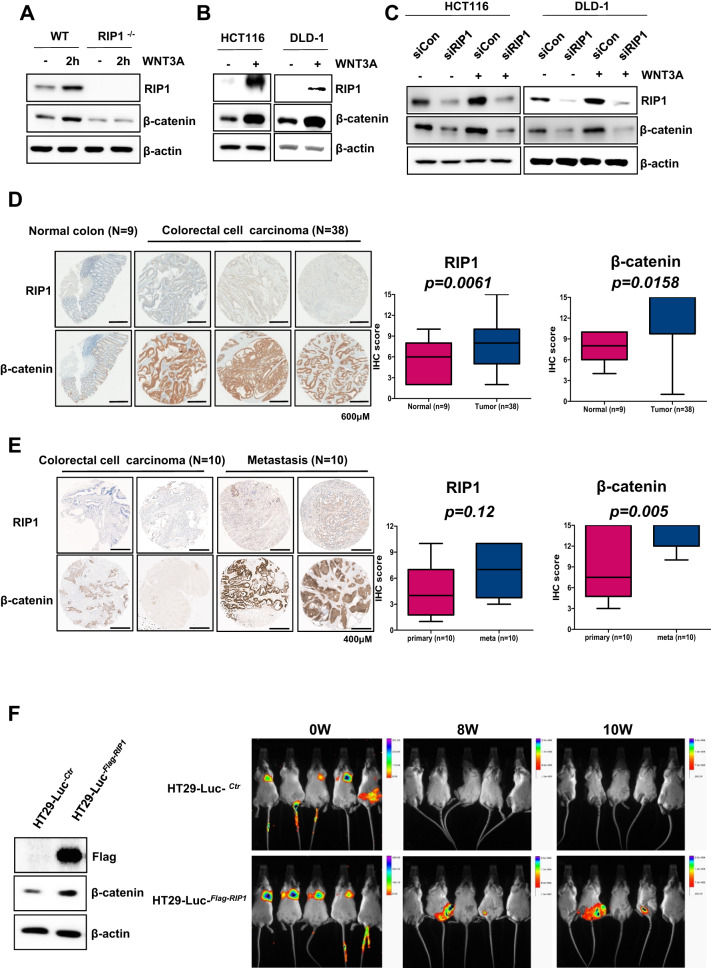
WNT3A upregulates RIP1 expression, resulting in β-catenin induction in CRC in vitro and in vivo. **A** IB analysis of RIP1 and β-catenin levels in wild-type and *RIP1*^-/-^ MEFs. **B** IB analysis of RIP1, β-catenin, and β-actin in HCT116 and DLD-1 cells. **C** IB analysis of RIP1, β-catenin, and β-actin in HCT116 and DLD-1 cells transfected with mock *control* (*Con*) siRNA or *RIP1* (si*RIP1*), followed by WNT3A treatment. **D** IHC analysis of a tumor microarray (CDA3) treated with antibodies against RIP1 and β-catenin. Upper panel: Representative images of normal colorectal tissues and primary CRC tissues. Lower panel: Correlation plot of RIP1 and β-catenin score quantification. **E** IHC analysis of a second tumor microarray (CO952a) treated with antibodies against RIP1 and β-catenin. Upper panel: Representative images of primary and metastatic CRC tissues. Lower panel: correlation plot of RIP1 and β-catenin score quantification. Boxes and whiskers indicate minimum-to-maximum percentiles. Center line, median value; upper box limit, 75% percentile; lower box limit, 25% percentile; whiskers, minimum or maximum values. **F** The expression levels of Flag and β-catenin in the HT29-Luc^*-Ctr*^ and HT29-Luc^*-RIP1*^ cells were examined by immunoblotting analysis. β-Actin was used as the loading control. Mice were separated into two groups: The control group was tail-vein injected with HT29-Luc^-*Ctr*^, and the experimental group was tail-vein injected with HT29-Luc^-*RIP1*^ cells.

Application of WNT3A (100 ng/mL) to wild-type (wt) or *RIP1*^*-/-*^ mouse embryonic fibroblasts (MEFs) for 2 h increased the level of β-catenin in wtMEFs, but not *RIP1*^*-/-*^ MEFs (Fig. 1A). *RIP1*^*-/-*^ MEFs showed relatively weak β-catenin expression in the mock control and WNT3A treatment groups. To further investigate the effect of WNT3A on RIP1 expression, we applied WNT3A to the HCT116 and DLD-1 CRC cell lines. We observed that the expression levels of endogenous RIP1 and β-catenin were increased simultaneously in both cell lines compared to the mock controls (Fig. 1B). To begin assessing where RIP1 acts in the WNT signaling pathway, we transfected HCT116 and DLD-1 cells with *control* siRNA or si*RIP1* for 24 h and then applied WNT3A. *RIP1* siRNA-transfected HCT116 and DLD-1 cells showed significantly decreased β-catenin expression (Fig. 1C). These results suggest that RIP1 might be a novel WNT signaling component located between WNT3A and β-catenin, and that WNT3A-induced β-catenin expression might be regulated by RIP1. We also performed immunofluorescence assays to demonstrate co-localization of RIP1 and β-catenin with or without WNT3A treatment (Supplementary Fig. 1). Unfortunately, we did not show dramatically translocation of β-catenin into nuclei in these experiments, but showed intracellular co-localization of RIP1 and β-catenin. In additionally, we also performed transfection of GFP-RIP1, and then observed co-localization of RIP1 and β-catenin (Supplementary Fig. 2). To assess the potential clinical significance of this finding, we performed IHC using a human CRC tissue microarray (TMA; CDA3) consisting of primary CRC tissues (*n* = 38) and normal colorectal tissues (*n* = 9) to determine the expression patterns of RIP1 and β-catenin (Fig. 1D). Quantitative IHC analyses revealed that RIP1 and β-catenin expression levels were significantly higher in primary CRC specimens than in normal colorectal tissues. Furthermore, IHC of RIP1 and β-catenin using a CO952a TMA consisting of primary CRC tissues (*n* = 10) and metastatic tissues (*n* = 10), such as lymph nodes (LN), ovaries, and lungs, indicated that the expression levels of RIP1 and β-catenin were elevated in metastatic tissues (Fig. 1E). In this experiment, β-catenin expression showed a significant increase, while RIP1 expression showed an increasing tendency. To study whether RIP1 could stimulate metastasis, we performed an in vivo animal metastasis experiment using stable HT29-Luc-^*Ctr*^ and HT29-Luc-^*RIP1*^ transfectants (Fig. 1F). Among NOD/SCID mice given a tail-vein injection of the transfectants, HT29-Luc-^*RIP1*^-injected mice showed luminescence in the body trunk at 8 weeks post-injection, indicating metastasis. These results suggest that RIP1 may increase CRC metastasis in vivo via WNT signaling.

### RIP1 increases β-catenin stability by direct binding and ubiquitination regulation

**Fig. 2:**
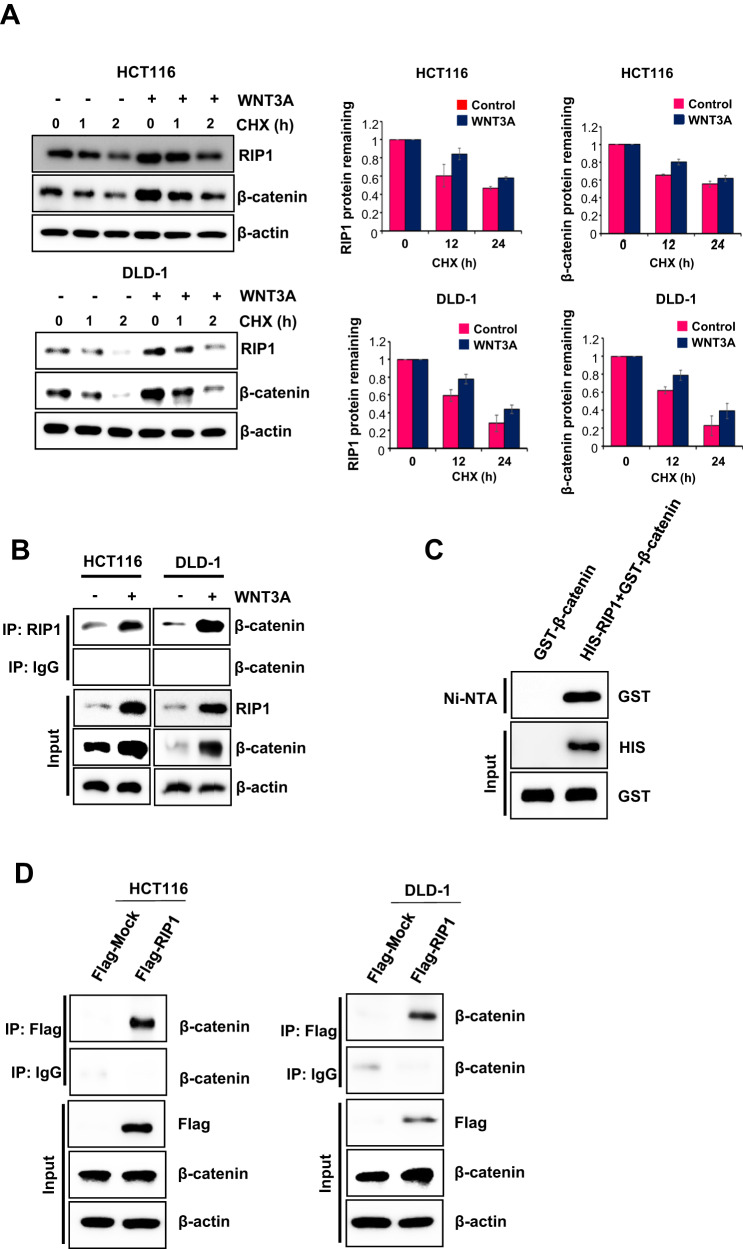
RIP1 increases β-catenin expression and stability. **A** Expression of RIP1 and β-catenin in HCT116 and DLD-1 cells. Left panels: Expression of RIP1 and β-catenin. Right panels: Graph of band densities of RIP1 and β-catenin. **B** IP assay was performed with HCT116 and DLD-1 cells treated with or without WNT3A. **C** In vitro binding assay of HIS-tagged RIP1 with GST-fused β-catenin. The detailed protocol is described in the Materials and Methods. **D** IP analyses were performed with HCT116 and DLD-1 cells transiently transfected with Flag-*Mock (pcDNA3.1)* or Flag-*RIP1* plasmids.

**Fig. 3:**
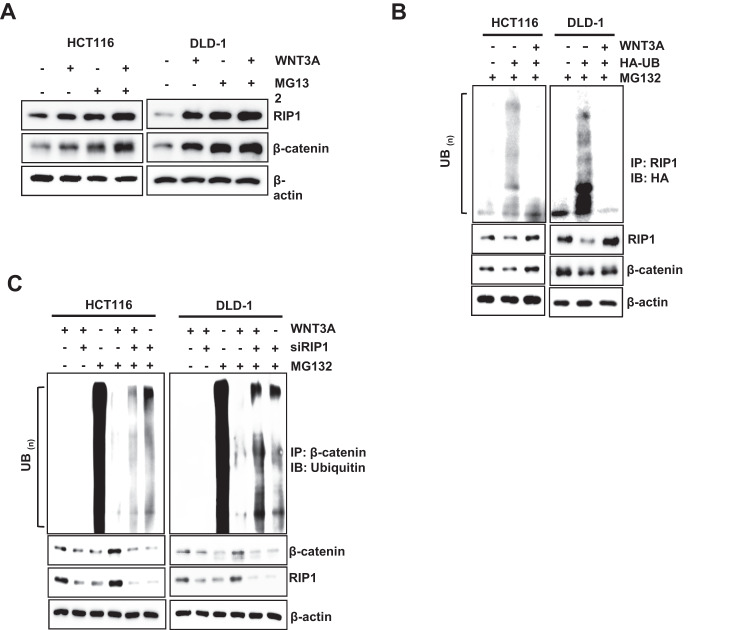
RIP1 increases β-catenin expression and stability dependent on the ubiquitination. **A** HCT116 and DLD-1 cells were treated with WNT3A for 2 h, followed by exposure to a MG132 for 6 h. IB analysis of RIP1 and β-catenin protein expression levels was performed. **B** Ubiquitylation assay was performed with HCT116 and DLD-1 cells transfected with or without HA-UB and then treated with MG132. **C** Ubiquitylation assay performed with HCT116 and DLD-1 cells transfected with siRNAs targeting *RIP1* (si*RIP1*) or control siRNA followed by treatment with WNT3A and MG132.

**Fig. 4:**
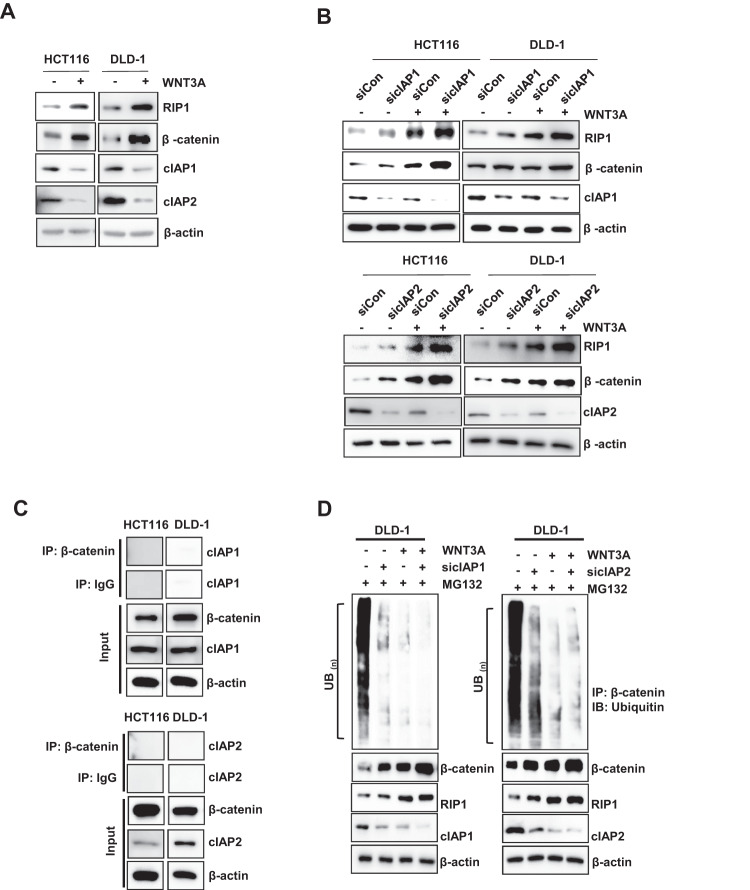
cIAP1/2 may regulate the expression and stability of β-catenin by controlling RIP1 ubiquitination. **A** HCT116 and DLD-1 cells were treated with WNT3A for 2 h and IB analysis for detection of RIP1, β-catenin, cIAP1, and cIAP2 was performed. β-Actin was used as the loading control. **B** IB analysis of RIP1 and β-catenin in HCT116 and DLD-1 cells transfected with siRNAs against *cIAP1* (si*cIAP1*) or *cIAP2* (si*cIAP2*) or *control* siRNA (si*Con*), followed by treatment with WNT3A. **C** HCT116 and DLD-1 cells were immunoprecipitated with β-catenin antibody, and then IB analysis was performed with anti-cIAP1 and cIAP2. **D** Ubiquitylation assay performed with DLD-1 cells transfected with si*cIAP1*, si*cIAP2* or si*Con*, followed by sequential treatment with WNT3A with or without 10 μM MG132.

To identify the role of RIP1 in the upregulation of β-catenin following WNT3A treatment, we treated HCT116 and DLD-1 cells with WNT3A. The addition of the de novo protein synthesis inhibitor CHX (100 μg/mL) revealed that the half-lives of RIP1 and β-catenin increased in the presence of WNT3A (Fig. 2A). We additionally performed western blotting assays to detect half-lives of RIP1 and β-catenin at 5 time points − 0, 3, 6, 12, and 24 h - after WNT3A treatment in HCT116 (Supplementary Fig. 3). These results also indicated WNT3A treatment could increase half-lives of RIP1 and β-catenin. We also investigated whether WNT3A could induce the interaction between RIP1 and β-catenin. IP revealed that RIP1 interacted with β-catenin after addition of WNT3A (Fig. 2B). Additionally, IP assay for β-catenin and the IB assay for RIP1 in HCT116 and DLD-1 also were performed (Supplementary Fig. 4). These results confirmed the binding of β-catenin and RIP1 in condition of WNT3A treatment. We performed in vitro binding assays to investigate whether HIS-RIP1 and GST-β-catenin could directly bind to each other and observed that RIP1 directly interacts with β-catenin in vitro (Fig. 2C). To confirm the binding between RIP1 and β-catenin in the absence of WNT, HCT116 and DLD-1 cells transiently transfected with Flag-Mock vector or Flag-RIP1 were used for IP with anti-Flag, followed by IB with anti-β-catenin antibodies (Fig. 2D). These results additionally demonstrated that RIP1 interacts directly with β-catenin in CRC cells. These results demonstrate that the stability of β-catenin is increased by its interaction with RIP1. To determine the mechanism responsible for regulating the half-life of RIP1 and β-catenin, we treated HCT116 and DLD-1 cells with MG132. WNT3A-induced induction of RIP1 and β-catenin was further increased by MG132 treatment, indicating that ubiquitination might be blocked by WNT3A (Fig. 3A). To determine the ubiquitination level of RIP1 under the influence of WNT3A, HCT116, and DLD-1 cells were treated with WNT3A and MG132. We observed that ubiquitination of RIP1 was significantly decreased following WNT3A treatment (Fig. 3B). Next, we used si*RIP1* to evaluate the effect of RIP1 on β-catenin ubiquitination in HCT116 and DLD1 cells. Previous studies have found that, in the absence of WNT stimulation, β-catenin is phosphorylated by a destruction complex that includes Axin, CK1, GSK3, and APC. Phosphorylated β-catenin is ubiquitinated by E3 ligase β-TrCP and degraded by the 26S proteasome. In contrast, under WNT stimulation, the destruction complex binds to the activated Wnt receptor, which leads to inhibition of the phosphorylation and ubiquitination of β-catenin and its subsequent translocation into the nucleus [25, 26]. Intriguingly, the WNT3A-medicated decrease in β-catenin ubiquitination was partially rescued by si*RIP1* (Fig. 3C). These data suggested that RIP1 specifically increased β-catenin stability by inhibiting its ubiquitination. To assess the potential regulatory mechanism of RIP1 on Wnt/β-catenin signaling, we assessed whether cIAP1/2 could regulate the interaction between RIP1 and β-catenin. Previous studies have indicated that cIAP1/2 possesses ubiquitin-protein isopeptide ligase (E3) activity in its RING domains, which facilitates constitutive RIP1 ubiquitination in cancer cells [27, 28]. We found that WNT3A treatment decreased endogenous cIAP1/2 levels and increased RIP1 and β-catenin levels (Fig. 4A). To further determine whether cIAP1/2 could regulate β-catenin stability, we used siRNAs against *cIAP1/2* and performed immunoblotting (IB) for RIP1 and β-catenin. Knockdown of cIAP1/2 upregulated the expression of RIP1 and β-catenin in WNT3A cells (Fig. 4B). As previous studies have shown that RIP1 interacts directly with cIAP1/2 proteins [27], we used an IP assay to test whether β-catenin interacts directly with cIAP1/2. We found that β-catenin did not directly interact with cIAP1/2 (Fig. 4C). Further β-catenin ubiquitination assay performed using *cIAP1/2* siRNAs revealed that downregulation of cIAP1/2 could decrease β-catenin ubiquitination with or without WNT3A (Fig. 4D). Together, these results suggest that the E3 ligase activity of cIAP1/2 may regulate the expression and stability of β-catenin by controlling RIP1 ubiquitination.

### WNT3A induced-RIP1 upregulation increases cell migration and invasion via EMT induction

**Fig. 5:**
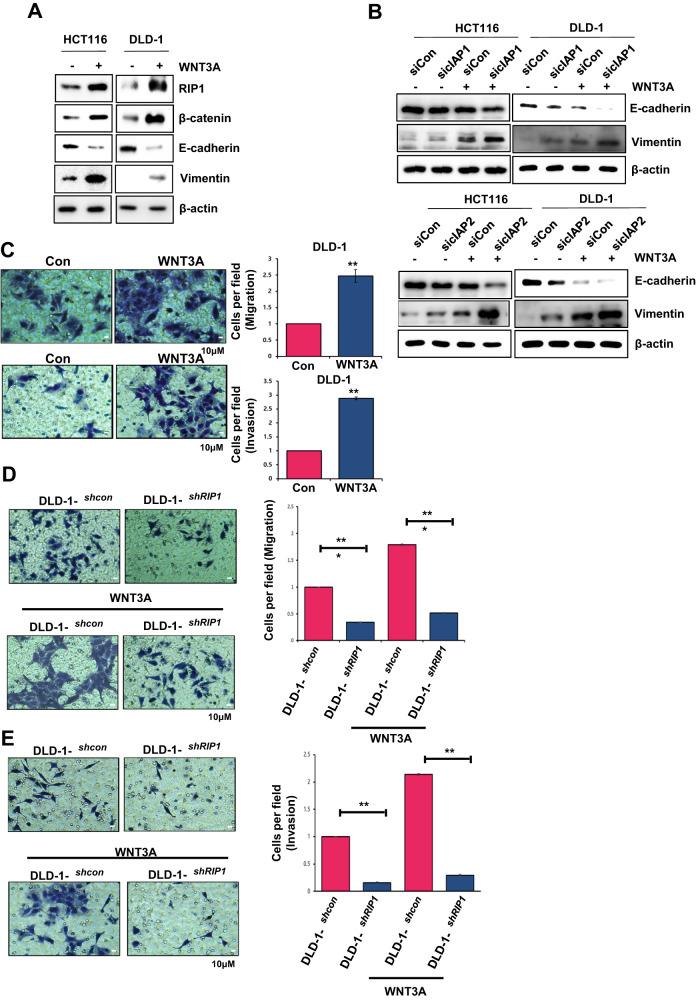
WNT3A-induced RIP1 expression increases the migration and invasiveness of CRC cells via EMT enhancement. **A** The levels of EMT markers were determined via IB analysis. **B** Detection of EMT markers by IB analysis was performed in HCT116 and DLD-1 cells transfected with siRNAs targeting *cIAP1* (si*cIAP1*) or *cIAP2* (si*cIAP2*) or with *control* siRNA (si*Con*). **C** Migration and invasion assays were performed with DLD-1 cells pre-treated with WNT3A for 48 h. Upper panels: Migration assay images (Left) and quantification graph (Right). Lower panels: invasion assay images (Left) and quantification graph (Right). **D** Migration assay with stable transfectant DLD-1 cell lines (DLD-1^*con*^ and DLD-1^*shRIP1*^) treated with or without WNT3A for 48 h. Left panels: Migration assay images. Right panel: Quantification graph. **E** Invasion assay with DLD-1^*con*^ and DLD-1^*shRIP1*^ treated with or without WNT3A for 48 h. Left panels: Invasion assay images. Right panel: quantification graph.

We investigated physiological changes in WNT3A-treated HCT116 and DLD-1 cells. IB analysis revealed that EMT was induced by WNT3A treatment, as evidenced by an increase in vimentin and a decrease in E-cadherin, accompanied by an increase in RIP1 and β-catenin (Fig. 5A). To determine whether cIAP1 or cIAP2 contributes to EMT induction, we used *cIAP1/2* siRNAs and performed IB. Indeed, the co-application of WNT3A and *cIAP1/2* siRNAs clearly induced EMT (Fig. 5B). Many previous studies have shown that EMT can promote invasion and migration of cancer cells [29–31]. We performed migration and invasion assays using a Transwell system and found that WNT3A treatment increased the migration and invasion abilities of DLD-1 cells by more than 2-fold (Fig. 5C). *RIP1*-knockdown DLD-1 cells (DLD-1^*shRIP1*^) exhibited a critical decrease in migration and invasion, to <2-fold that of DLD-1^*shcon*^. WNT3A treatment of DLD-1^*shRIP1*^ cells intensified this decrease by more than 3-fold (Fig. 5D, E). These results imply that RIP1 in CRC cells could increase migration and invasion via EMT in combination with WNT signaling.

### RIP1 interrupts the β-catenin—β-TrCP interaction

**Fig. 6:**
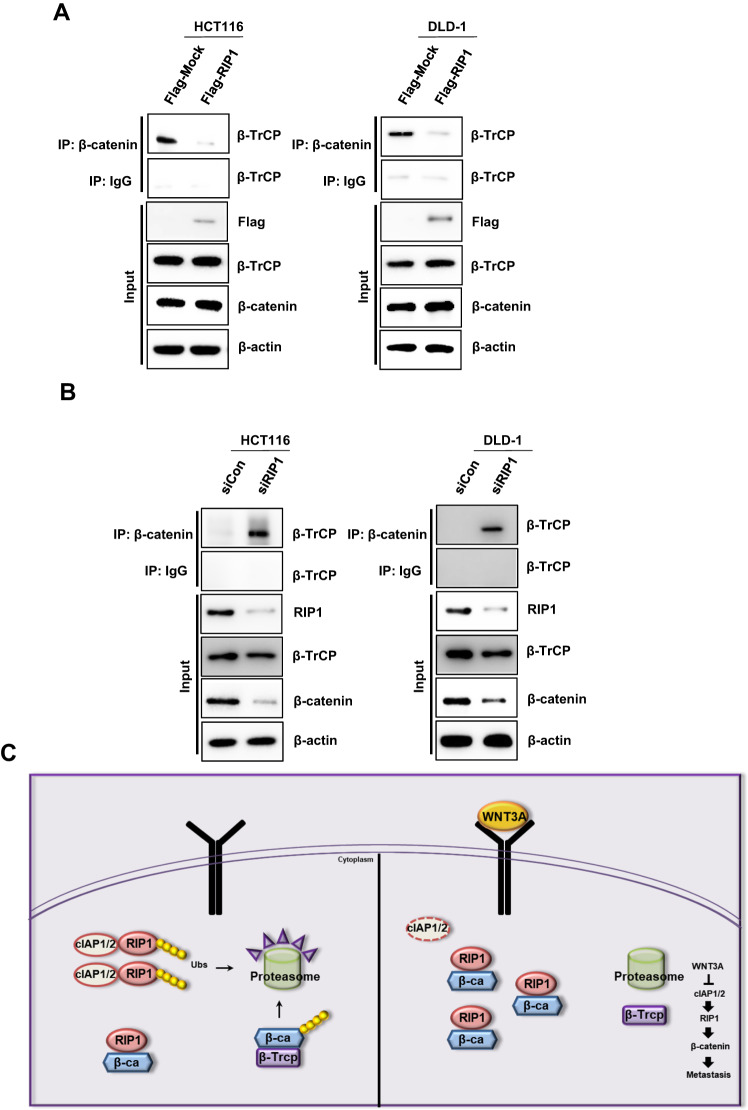
Stably expressed RIP1 directly binds β-catenin and disrupts the β-catenin―β-TrCP complex. **A** IP analyses were performed with HCT116 and DLD-1 cells transiently transfected with Flag-*Mock (pcDNA3.1)* or Flag-*RIP1* plasmids. **B** IP analyses were performed with HCT116 and DLD-1 cells transiently transfected with mock *control* (*Con*) *siRNA* or *RIP1* (si*RIP1*). **C** Proposed model for the role of RIP1 in CRC metastasis.

We used this system to assess the regulation of β-catenin by RIP1. β-catenin is phosphorylated on serine-33 and −37 by Gsk3 and on serine-45 and threonine-41 by CK1 in the absence of the WNT ligand [32]. Phosphorylated β-catenin is recognized and bound by E3 ubiquitin ligase β-transducin repeat-containing protein (β-TrCP). WNT abrogates the recruitment of β-TrCP to β-catenin and sequentially blocks β-catenin ubiquitination [25, 26]. To assess whether RIP1 affects this process, we performed IP with an anti-β-catenin antibody, followed by IB with an anti-β-TrCP antibody. The results revealed that RIP1 decreased the β-catenin–β-TrCP interaction without altering the protein levels of β-catenin and β-TrCP (Fig. 6A). Also, we transfected HCT116 and DLD-1 cells with *control* siRNA or si*RIP1* for 24 h. We performed IP with an anti-β-catenin antibody, followed by IB with an anti-β-TrCP antibody. *RIP1* siRNA-transfected HCT116 and DLD-1 cells showed significantly increased β-catenin-β-TrCP interaction (Fig. 6B). Taken together, our results enabled us to construct a schematic signaling model for the proposed role of RIP1 in promoting CRC metastasis (Fig. 6C). In this model, WNT3A treatment induced cIAP1/2 degradation, enabling RIP1 and β-catenin to bind and stabilize one another, leading to increased protein levels. This binding of RIP1 and β-catenin also stimulates the dissociation of the β-catenin–β-TrCP complex, implying that the binding affinity of the β-catenin–β-TrCP complex is weaker than that between RIP1 and β-catenin. Finally, these intracellular changes might promote CRC metastasis in patients, suggesting novel strategies for treating CRC.

## Discussion

RIP1 expression levels are associated with the type of cancer. In CRC, in particular, RIP1 is upregulated and promotes cell proliferation via interacting with mitochondrial Ca^2+^ uniporter (MCU) to increase mitochondrial Ca^2+^ uptake and energy metabolism [33]. Our present study provided novel insights into the role of RIP1 in CRC pathogenesis by showing that it mediates the WNT signaling pathway. First, our data suggest that RIP1 may be a novel marker for the prognosis of CRC metastasis in personalized therapy. We found that RIP1 could contribute to the induction of metastasis in CRC specimens. TMA indicated that RIP1 expression is upregulated in CRC primary cancer tissues, and that the levels of RIP1 and β-catenin are higher in metastatic tissues of CRC patients than in their CRC primary cancer tissues (Fig. 1D, E). These results imply that a higher RIP1 expression level could promote the metastasis and cellular proliferation of CRC. Second, because WNT signaling alterations are seen in almost all CRC specimens [34, 35], we examined the effect of WNT3A on RIP1 expression in CRC cell lines as a means to begin identifying the mechanisms underlying RIP1-regulated metastasis. Previous work showed that mutational inactivation of the tumor suppressor, adenomatous polyposis coli (APC), which is a major component of β-catenin destruction complex, is an initiating step in sporadic and familial CRC [36]. Moreover, hyperactivation of WNT signaling by genetic mutations or constitutive WNT ligand treatment was shown to stimulate CRC malignancy parameters, such as EMT, migration, invasion, and metastasis [37]. We used WNT3A as ligand to stimulate Wnt/β-catenin signaling because Wnt3a has been reported to promote proliferation, differentiation, migration, and self-renewal in numerous solid cancers, including CRC [38]. Consistent with the previous reports, we observed that the endogenous levels of RIP1 and β-catenin increased simultaneously when CRC cells were treated with WNT3A (Fig. 1B). To determine the locations of RIP1 and β-catenin in Wnt/β-catenin signaling, we applied *RIP1* siRNA. This treatment significantly decreased the expression of endogenous β-catenin in our system (Fig. 1C), implying that RIP1 is located upstream of β-catenin. IP assays performed using RIP1 or FLAG antibodies showed that both endogenous and exogenous RIP1 could directly interact with β-catenin (Figs. 2B, D). In vitro binding assays performed using recombinant RIP1 or β-catenin proteins indicated that HIS-tagged RIP1 could bind GST-tagged β-catenin in an in vitro system (Fig. 2C). These results indicate that RIP1 directly interacts with β-catenin, and that the WNT3A-mediated upregulation of RIP1 might increase the stability of β-catenin via direct binding.

We found that RIP1 inhibited the ubiquitination of β-catenin to decrease its proteasomal degradation (Fig. 3C). β-catenin engages in various functions in multiple cellular events and human diseases. The core aspect of Wnt/β-catenin signaling is the balance between the phosphorylation/dephosphorylation and degradation of β-catenin. The destruction complex involved in Wnt/β-catenin signaling phosphorylates β-catenin and subsequently degrades it via the ubiquitin-proteasome system. Various kinases contribute to protein phosphorylation in the Wnt/β-catenin signaling pathway. Among them, PP1 and PP2C stimulate Wnt/β-catenin signaling by dephosphorylating axin [39, 40]. PP2A is a Ser/Thr phosphatase whose regulatory subunit, PR553α, suppresses β-catenin phosphorylation. The PP2A regulator, Hsp105, decreases β-catenin degradation. Previous studies found that PP2A is overexpressed in various cancers and stimulates β-catenin accumulation [41, 42]. These studies found that stabilization of β-catenin is important in the tumorigenesis, progression, and prognosis of various cancers. In particular, a constitutively activated form of β-catenin involving a mutation in exon 3 is thought to regulate the occurrence of hereditary non-polyposis CRC [43]. We thus hypothesized that RIP1 might be an important and novel upstream effector of β-catenin in canonical WNT signaling. Interestingly, WNT3A also decreased the ubiquitination of RIP1 (Fig. 3B), suggesting that RIP1 ubiquitination might decrease the stability of β-catenin. We further found that WNT3A decreased the expression of cIAP1/2. This E3 ligase is known to modify RIP1 by attaching various ubiquitin chains, such as M1, K11, and K63, in complex I of TNFR1 signaling [27]. This modified RIP1 functions to recruit other ubiquitin-binding proteins, such as TAB2/3 and NEMO [44, 45]. However, our present results indicate that WNT3A decreases the ubiquitination of RIP1 and β-catenin and upregulates their expression levels, while downregulating the expression of cIAP1/2 (Fig. 4). Downregulation of cIAP1/2 with *cIAP1/2* siRNA treatment also decreases the ubiquitination and upregulates the expression of RIP1 and β-catenin. These results are consistent with previous reports that cIAP1/2 can directly conjugate diverse ubiquitin chains to RIP1 to recruit other proteins [27], the other way, we find cIAP1/2 E3 ligase functions negative regulator to WNT3A-mediated RIP1 and β-catenin binding in this study. In addition to the ability of cIAP1/2 to regulate RIP1, we also found that RIP1 might act as a strong binding competitor of the β-catenin-targeted E3 ligase, β-TrCP, to inhibit β-catenin ubiquitination (Fig. 6A and B) [25, 26]. These results indicate that the dissociation of bound ubiquitin is necessary for the binding of RIP1 and β-catenin, which precedes WNT signaling (Fig. 6C).

Taken together, our results suggest that RIP1 may be a novel EMT inducer that promotes metastasis in CRC by decreasing the ubiquitination and increasing the stability of β-catenin in WNT/β-catenin signaling. This novel role of RIP1 in WNT signaling may suggest that crosstalk between TNF and WNT signaling may modulate CRC malignancy. Also, given that many researchers are seeking to develop novel drugs targeting RIP1 and WNT signaling, our study could support the development of new combinatorial therapeutic strategies for regulating CRC by controlling the two major inflammatory pathways of TNF and Wnt/β-catenin signaling.

### Supplementary information

Supplementary Figures

Supplementary Figure legends

supplementary western image

## Data Availability

All data in the main text or the supplementary materials are available from the corresponding authors on request.

## References

[1] Rawla P, Sunkara T, Barsouk A (2019). Epidemiology of colorectal cancer: incidence, mortality, survival, and risk factors. Prz Gastroenterol.

[2] Pretzsch E, Bösch F, Neumann J, Ganschow P, Bazhin A, Guba M (2019). Mechanisms of metastasis in colorectal cancer and metastatic organotropism: hematogenous versus peritoneal spread. J Oncol.

[3] Vatandoust S, Price TJ, Karapetis CS (2015). Colorectal cancer: metastases to a single organ. World J Gastroenterol.

[4] Yue B, Liu C, Sun H, Liu M, Song C, Cui R (2018). A positive feed-forward loop between LncRNA-CYTOR and Wnt/β-catenin signaling promotes metastasis of colon cancer. Mol Ther.

[5] Zhang M, Miao F, Huang R, Liu W, Zhao Y, Jiao T (2018). RHBDD1 promotes colorectal cancer metastasis through the Wnt signaling pathway and its downstream target ZEB1. J Exp Clin Cancer Res.

[6] Li Q, Lai Q, He C, Fang Y, Yan Q, Zhang Y (2019). RUNX1 promotes tumour metastasis by activating the Wnt/β-catenin signalling pathway and EMT in colorectal cancer. J Exp Clin Cancer Res.

[7] Wang Z, Zhao T, Zhang S, Wang J, Chen Y, Zhao H (2021). The Wnt signaling pathway in tumorigenesis, pharmacological targets, and drug development for cancer therapy. Biomark Res.

[8] Yang K, Wang X, Zhang H, Wang Z, Nan G, Li Y (2016). The evolving roles of canonical WNT signaling in stem cells and tumorigenesis: implications in targeted cancer therapies. Lab Investig.

[9] Wang C, Zhang R, Wang X, Zheng Y, Jia H, Li H (2021). Silencing of KIF3B suppresses breast cancer progression by regulating EMT and wnt/β-catenin signaling. Front Oncol.

[10] Lomphithak T, Choksi S, Mutirangura A, Tohtong R, Tencomnao T, Usubuchi H (2020). Receptor-interacting protein kinase 1 is a key mediator in TLR3 ligand and Smac mimetic-induced cell death and suppresses TLR3 ligand-promoted invasion in cholangiocarcinoma. Cell Commun Signal.

[11] Vandenabeele P, Declercq W, Van Herreweghe F, Vanden, Berghe T (2010). The role of the kinases RIP1 and RIP3 in TNF-induced necrosis. Sci Signal.

[12] Kondylis V, Pasparakis M (2019). RIP kinases in liver cell death, inflammation and cancer. Trends Mol Med.

[13] Varfolomeev E, Vucic D (2022). RIP1 post-translational modifications. Biochem J.

[14] Christofferson DE, Li Y, Yuan J (2014). Control of life-or-death decisions by RIP1 kinase. Annu Rev Physiol.

[15] Zhu G, Chen X, Wang X, Li X, Du Q, Hong H (2014). Expression of the RIP-1 gene and its role in growth and invasion of human gallbladder carcinoma. Cell Physiol Biochem.

[16] Liu XY, Lai F, Yan XG, Jiang CC, Guo ST, Wang CY (2015). RIP1 kinase is an oncogenic driver in melanomaRIP1 in melanoma. Cancer Res.

[17] Zheng X-L, Yang J-J, Wang Y-Y, Li Q, Song Y-P, Su M (2020). RIP1 promotes proliferation through G2/M checkpoint progression and mediates cisplatin-induced apoptosis and necroptosis in human ovarian cancer cells. Acta Pharmacol Sin.

[18] Li C-Z, Jiang X-J, Lin B, Hong H-J, Zhu S-Y, Jiang L (2018). RIP1 regulates TNF-α-mediated lymphangiogenesis and lymphatic metastasis in gallbladder cancer by modulating the NF-κB-VEGF-C pathway. OncoTargets Ther.

[19] Kang A-R, Cho JH, Lee N-G, Song J-Y, Hwang S-G, Lee D-H (2020). RIP1 is a novel component of γ-ionizing radiation-induced invasion of non-small cell lung cancer cells. Int J Mol Sci.

[20] Kang A-R, Cho JH, Lee N-G, Kwon J-H, Song J-Y, Hwang S-G (2021). Radiation-induced IL-1β expression and secretion promote cancer cell migration/invasion via activation of the NF-κB–RIP1 pathway. Biochem Biophys Res Commun.

[21] Piao SG, Ding J, Lin XJ, Nan QY, Xuan MY, Jiang YJ (2022). Inhibition of RIP1-RIP3-mediated necroptosis attenuates renal fibrosis via Wnt3α/β-catenin/GSK-3β signaling in unilateral ureteral obstruction. PLoS ONE.

[22] Kang A-R, An H-T, Ko J, Kang S (2017). Ataxin-1 regulates epithelial–mesenchymal transition of cervical cancer cells. Oncotarget.

[23] Kang AR, Park SH, Lee S, Choi DY, Kim KP, Song HK (2015). A key lysine residue in the AXH domain of ataxin-1 is essential for its ubiquitylation. Biochim Biophys Acta (BBA)-Proteins Proteom.

[24] Kang A-R, An H-T, Ko J, Choi E-J, Kang S (2017). Ataxin-1 is involved in tumorigenesis of cervical cancer cells via the EGFR–RAS–MAPK signaling pathway. Oncotarget.

[25] Zhan T, Rindtorff N, Boutros M (2017). Wnt signaling in cancer. Oncogene.

[26] Zhang Y, Wang X (2020). Targeting the Wnt/β-catenin signaling pathway in cancer. J Hematol Oncol.

[27] Bertrand MJ, Milutinovic S, Dickson KM, Ho WC, Boudreault A, Durkin J (2008). cIAP1 and cIAP2 facilitate cancer cell survival by functioning as E3 ligases that promote RIP1 ubiquitination. Mol Cell.

[28] Annibaldi A, John SW, Berghe TV, Swatek KN, Ruan J, Liccardi G (2018). Ubiquitin-mediated regulation of RIPK1 kinase activity independent of IKK and MK2. Mol Cell.

[29] Son H, Moon A (2010). Epithelial-mesenchymal transition and cell invasion. Toxicol Res.

[30] Yeung KT, Yang J (2017). Epithelial–mesenchymal transition in tumor metastasis. Mol Oncol.

[31] Tsai JH, Yang J (2013). Epithelial–mesenchymal plasticity in carcinoma metastasis. Genes Dev.

[32] Nusse R, Clevers H (2017). Wnt/β-catenin signaling, disease, and emerging therapeutic modalities. Cell.

[33] Zeng F, Chen X, Cui W, Wen W, Lu F, Sun X (2018). RIPK1 binds MCU to mediate induction of mitochondrial Ca2+ uptake and promotes colorectal oncogenesisRIPK1 and MCU promote colorectal cancer progression. Cancer Res.

[34] Network CGA (2012). Comprehensive molecular characterization of human colon and rectal cancer. Nature.

[35] Novellasdemunt L, Foglizzo V, Cuadrado L, Antas P, Kucharska A, Encheva V (2017). USP7 is a tumor-specific WNT activator for APC-mutated colorectal cancer by mediating β-catenin deubiquitination. Cell Rep.

[36] Zhang L, Shay JW (2017). Multiple roles of APC and its therapeutic implications in colorectal cancer. J Natl Cancer Inst.

[37] Zhou H, Zhu L, Song J, Wang G, Li P, Li W (2022). Liquid biopsy at the frontier of detection, prognosis and progression monitoring in colorectal cancer. Mol Cancer.

[38] Guo W, Wang H, Li C (2021). Signal pathways of melanoma and targeted therapy. Signal Transduct Target Ther.

[39] Luo W, Peterson A, Garcia BA, Coombs G, Kofahl B, Heinrich R (2007). Protein phosphatase 1 regulates assembly and function of the β‐catenin degradation complex. EMBO J.

[40] Strovel ET, Wu D, Sussman DJ (2000). Protein phosphatase 2Cα dephosphorylates axin and activates LEF-1-dependent transcription. J Biol Chem.

[41] Zhang W, Yang J, Liu Y, Chen X, Yu T, Jia J (2009). PR55α, a regulatory subunit of PP2A, specifically regulates PP2A-mediated β-catenin dephosphorylation. J Biol Chem.

[42] Yu N, Kakunda M, Pham V, Lill JR, Du P, Wongchenko M (2015). HSP105 recruits protein phosphatase 2A to dephosphorylate β-catenin. Mol Cell Biol.

[43] Johnson V, Volikos E, Halford S, Sadat EE, Popat S, Talbot I (2005). Exon 3 β-catenin mutations are specifically associated with colorectal carcinomas in hereditary non-polyposis colorectal cancer syndrome. Gut.

[44] Witt A, Vucic D (2017). Diverse ubiquitin linkages regulate RIP kinases-mediated inflammatory and cell death signaling. Cell Death Differ.

[45] McCool K, Miyamoto S (2009). A PAR-SUMOnious mechanism of NEMO activation. Mol Cell.

