# Supplementary material for: A novel RIP1-mediated canonical WNT signaling pathway that promotes colorectal cancer metastasis via β -catenin stabilization-induced EMT
Source: Cancer Gene Ther. 2023 Jul 27;30(10):1403–13. doi: 10.1038/s41417-023-00647-6 (PMC10581897; doi:10.1038/s41417-023-00647-6)
Supplement: Supplementary file 1 — Supplementary Figures [file 41417_2023_647_MOESM1_ESM.pptx]

## Slide 1
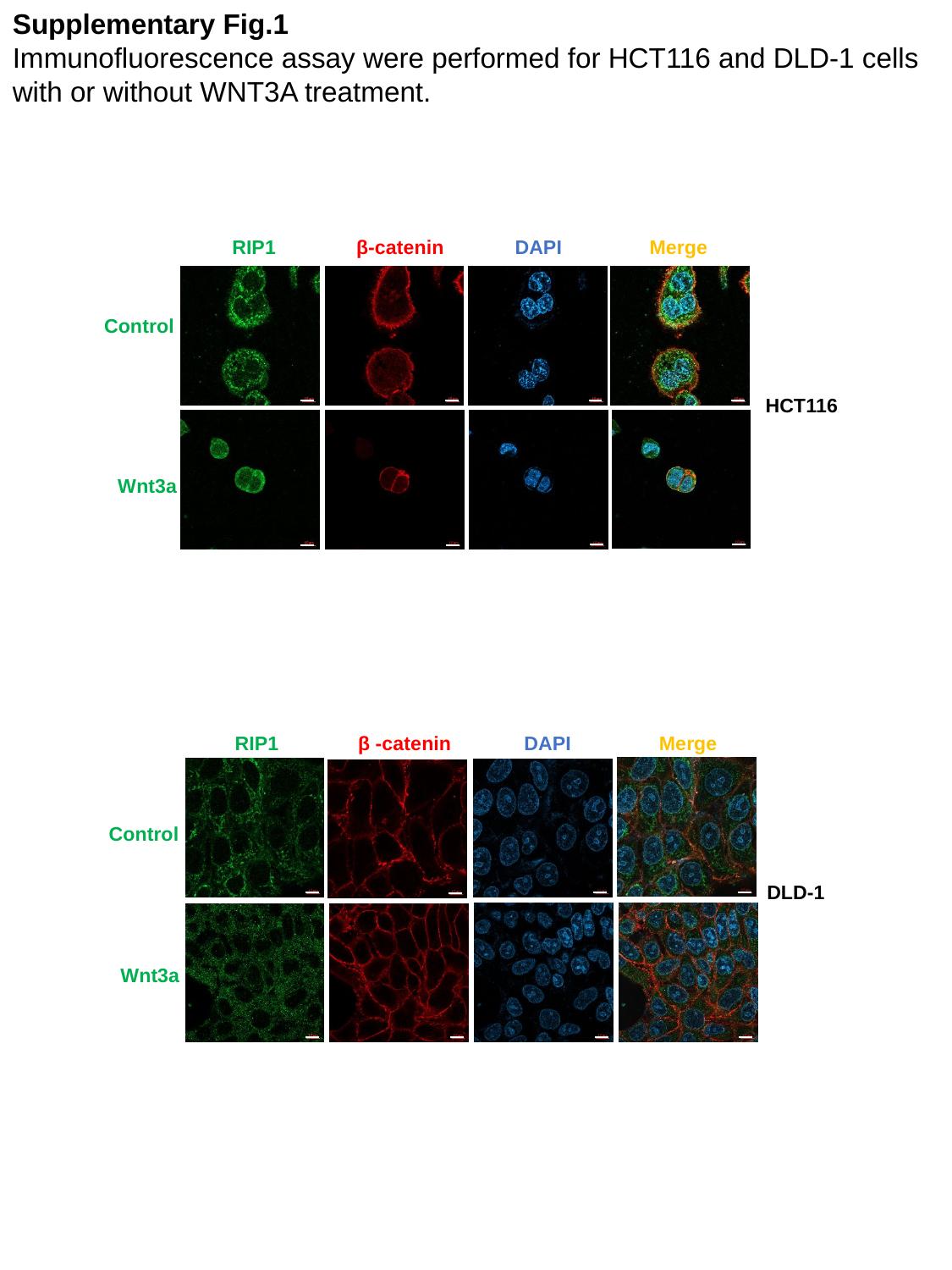

Supplementary Fig.1
Immunofluorescence assay were performed for HCT116 and DLD-1 cells with or without WNT3A treatment.
RIP1
β-catenin
DAPI
Merge
Control
HCT116
Wnt3a
RIP1
β -catenin
DAPI
Merge
Control
DLD-1
Wnt3a

## Slide 2
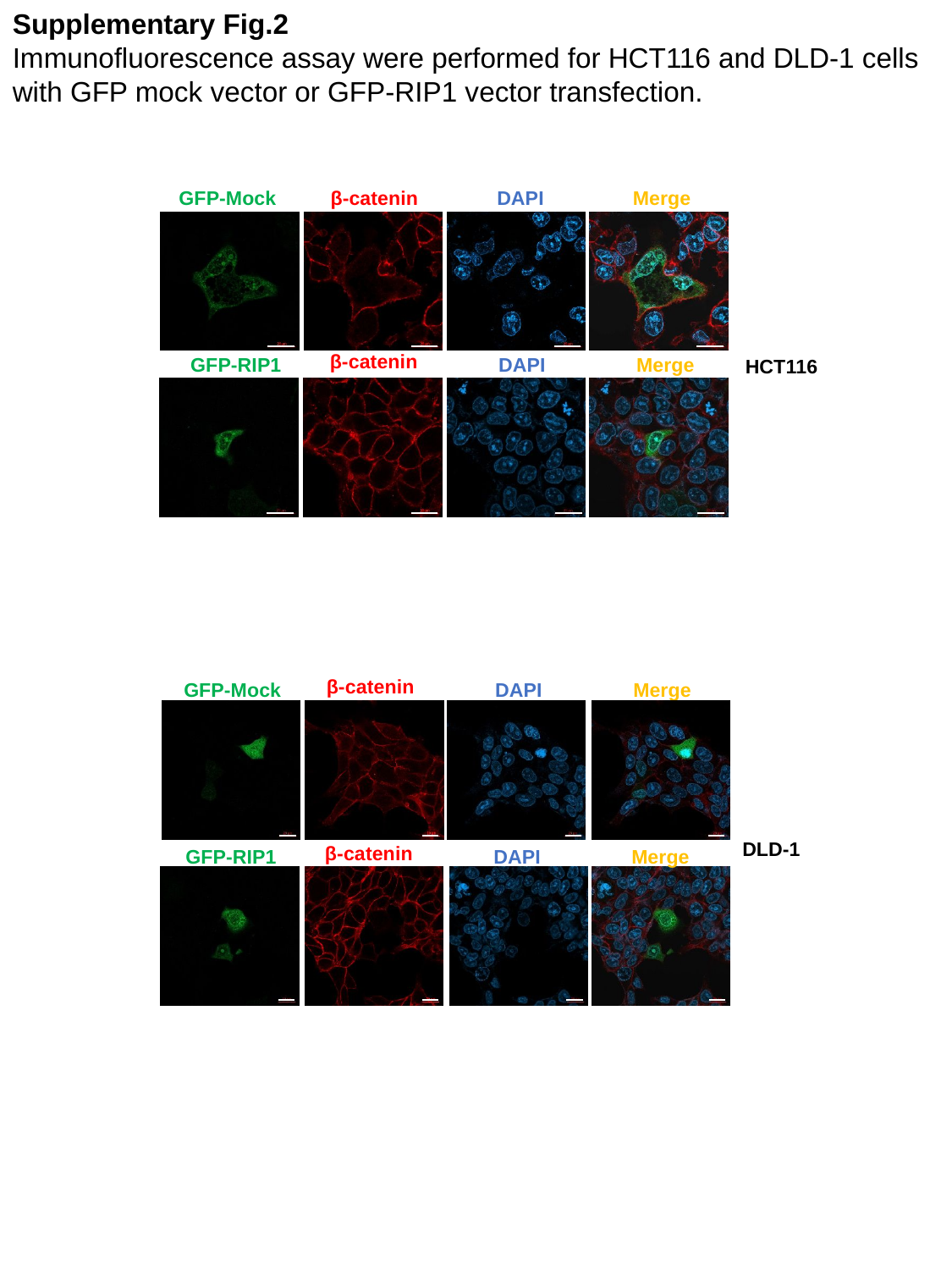

Supplementary Fig.2
Immunofluorescence assay were performed for HCT116 and DLD-1 cells with GFP mock vector or GFP-RIP1 vector transfection.
GFP-Mock
β-catenin
DAPI
Merge
HCT116
β-catenin
GFP-RIP1
DAPI
Merge
β-catenin
GFP-Mock
DAPI
Merge
DLD-1
β-catenin
GFP-RIP1
DAPI
Merge

## Slide 3
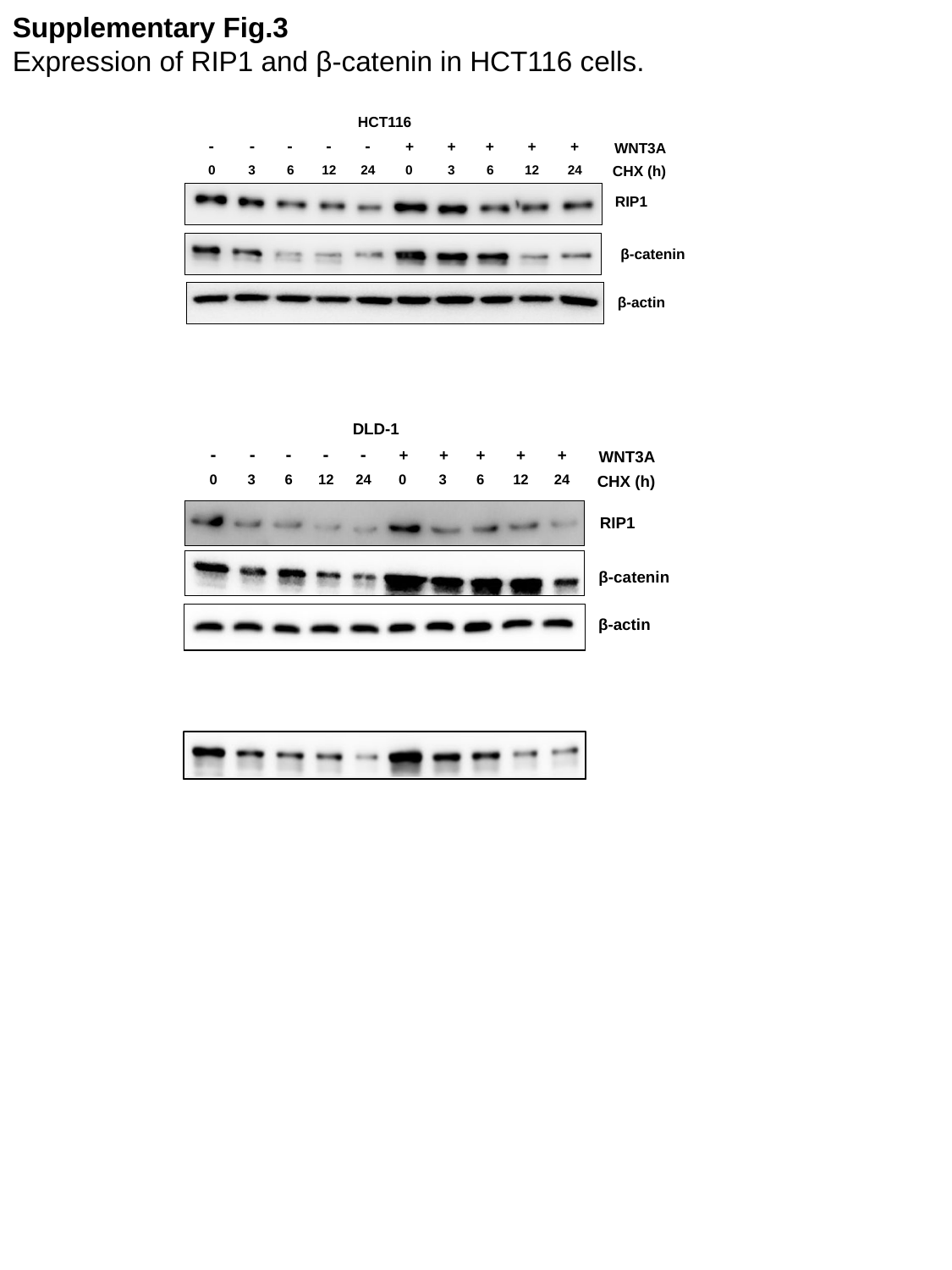

Supplementary Fig.3
Expression of RIP1 and β-catenin in HCT116 cells.
HCT116
 -
 -
 -
 -
 -
 +
 +
 +
 +
 +
WNT3A
0
3
6
12
24
0
3
6
12
24
CHX (h)
RIP1
β-catenin
β-actin
DLD-1
 -
 -
 -
 -
 -
 +
 +
 +
 +
 +
WNT3A
0
3
6
12
24
0
3
6
12
24
CHX (h)
RIP1
β-catenin
β-actin

## Slide 4
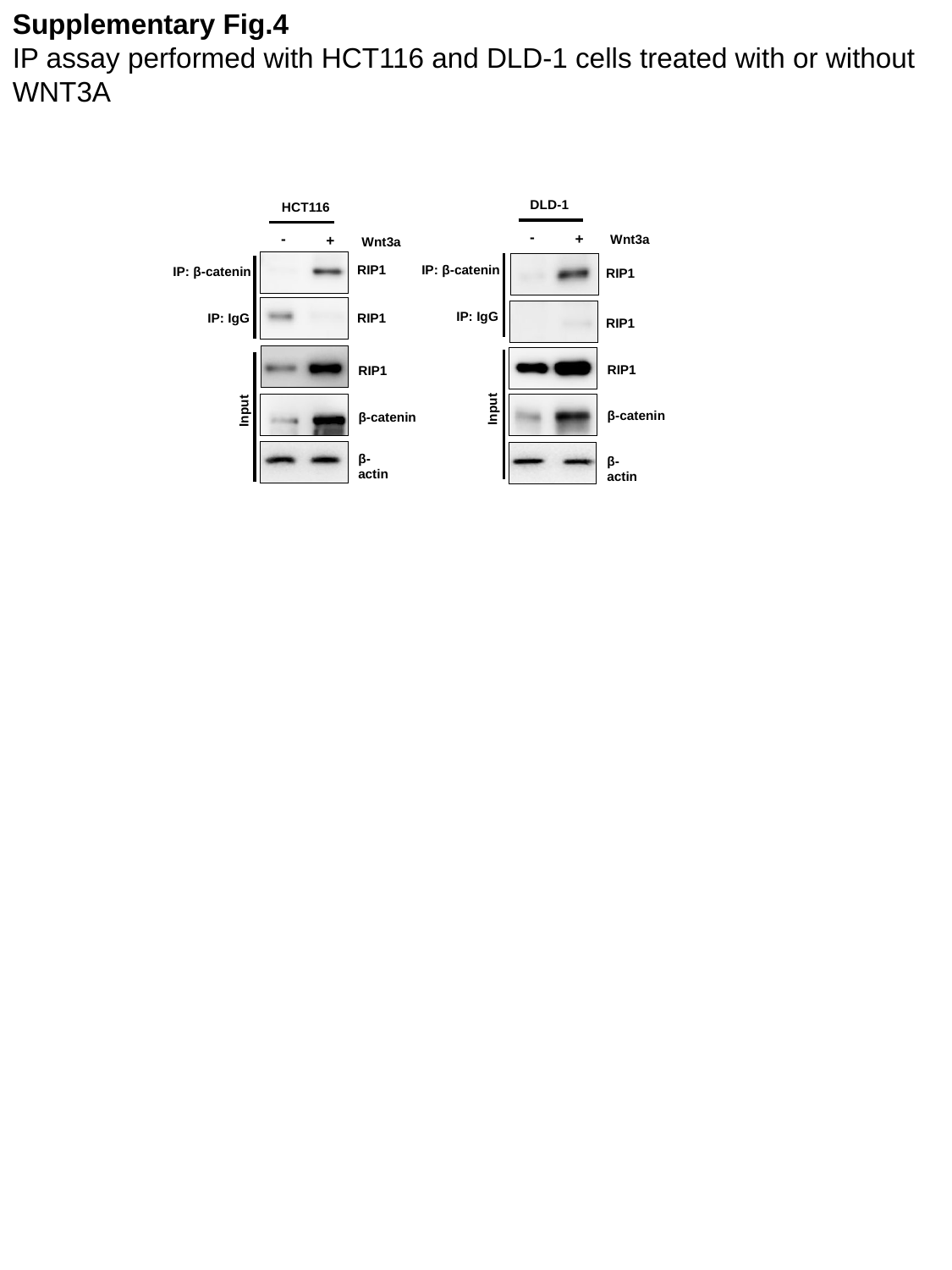

Supplementary Fig.4
IP assay performed with HCT116 and DLD-1 cells treated with or without WNT3A
DLD-1
HCT116
-
+
-
Wnt3a
+
Wnt3a
IP: β-catenin
RIP1
IP: β-catenin
RIP1
IP: IgG
RIP1
IP: IgG
RIP1
RIP1
RIP1
Input
Input
β-catenin
β-catenin
β-actin
β-actin
