# Supplementary material for: A novel RIP1-mediated canonical WNT signaling pathway that promotes colorectal cancer metastasis via β -catenin stabilization-induced EMT
Source: Cancer Gene Ther. 2023 Jul 27;30(10):1403–13. doi: 10.1038/s41417-023-00647-6 (PMC10581897; doi:10.1038/s41417-023-00647-6)
Supplement: Supplementary file 2 — Supplementary Figure legends [file 41417_2023_647_MOESM2_ESM.docx]

**Supplementary Figure legends**

**Supplementary Figure 1.** Immunofluorescence assay were performed for HCT116 and DLD-1 cells with or without WNT3A treatment. For immunofluorescent staining, HCT116 and DLD-1 cells grown on the surface of cover slides were serum-starved overnight followed by stimulation with 100 ng/ml WNT3A for 2 hours. After fixation, cells were lysed in 0.1% Triton X-100 for 5 min and incubated with an anti-RIP1 antibody (BD Biosciences) and anti-β-catenin (Alexa Fluor^®^ 594 conjugate, cell signaling, cat#70194) overnight, followed by an Alexa Fluor 488^®^-conjugated goat anti-mouse IgG (H + L) antibody (Invitrogen, cat# A-11029, 1: 50 dilution) for 2 h at room temperature. Nuclei were stained with DAPI mounting solution (Sigma Aldrich, St. Louis, MO, USA, DUO82040) and images of stained cells were acquired under a LSM800 confocal microscope (Carl Zeiss, Germany)

**Supplementary Figure 2**. Immunofluorescence assay were performed for HCT116 and DLD-1 cells with GFP mock vector or GFP-RIP1 vector transfection. Immunofluorescence Assay (IFA) were performed as followed. HCT116 and DLD-1 cells seeded into 12-well chamber slides (Nunc Inc., IL, USA) were GFP mock-transfected or transfected with GFP-RIP1 for 24 h. Briefly, cells were washed with PBS and fixed with 4% paraformaldehyde for 20 min (Supplementary Fig 3). After fixation, cells were lysed in 0.1% Triton X-100 for 5 min and incubated with anti-β-catenin (Alexa Fluor® 594 conjugate, cell signaling, cat#70194) overnight. Nuclei were stained with DAPI mounting solution (Sigma Aldrich, St. Louis, MO, USA, DUO82040) and images of stained cells were acquired under a LSM800 confocal microscope (Carl Zeiss, Germany)

**Supplementary Figure 3.** Expression of RIP1 and β-catenin in HCT116 cells.HCT116 cells were serum-starved for 16 h and then treated with or without WNT3A (100 ng/ml) for 2 h. After 24 h, the cells were treated with 100 μg/mL cycloheximide (CHX; Sigma-Aldrich, St. Louis, MO, USA) to inhibit protein synthesis. Cycloheximide-treated cells were collected at the indicated time points (0, 3, 6, 12, and 24 h) and processed for IB using antibodies against RIP1, β-catenin, and β-actin.

**Supplementary Figure 4.** IP assay performed with HCT116 and DLD-1 cells treated with or without WNT3A. HCT116 and DLD-1 cells were transiently transfected with Flag-*Mock* (*pcDNA3.1*) or Flag-*RIP1*. After 48 h, cells were harvested and lysed on ice for 60 min in 0.7% NP-40 lysis buffer supplemented with protease inhibitors (10 µg/ml aprotinin, 10 µg/ml leupeptin, and 2 mM PMSF). The resulting lysates were centrifuged at 13,000×*g* for 20 min at 4°C, and the supernatants were incubated with the anti- β-catenin antibody at 4°C overnight. Protein G-Sepharose beads (GE Healthcare, Little Chalfont, UK) were then added, and bead-bound proteins were analyzed using sodium dodecyl sulfate (SDS)–polyacrylamide gel electrophoresis.
