# Supplementary material for: A novel RIP1-mediated canonical WNT signaling pathway that promotes colorectal cancer metastasis via β -catenin stabilization-induced EMT
Source: Cancer Gene Ther. 2023 Jul 27;30(10):1403–13. doi: 10.1038/s41417-023-00647-6 (PMC10581897; doi:10.1038/s41417-023-00647-6)
Supplement: Supplementary file 3 — supplementary western image [file 41417_2023_647_MOESM3_ESM.pptx]

## Slide 1
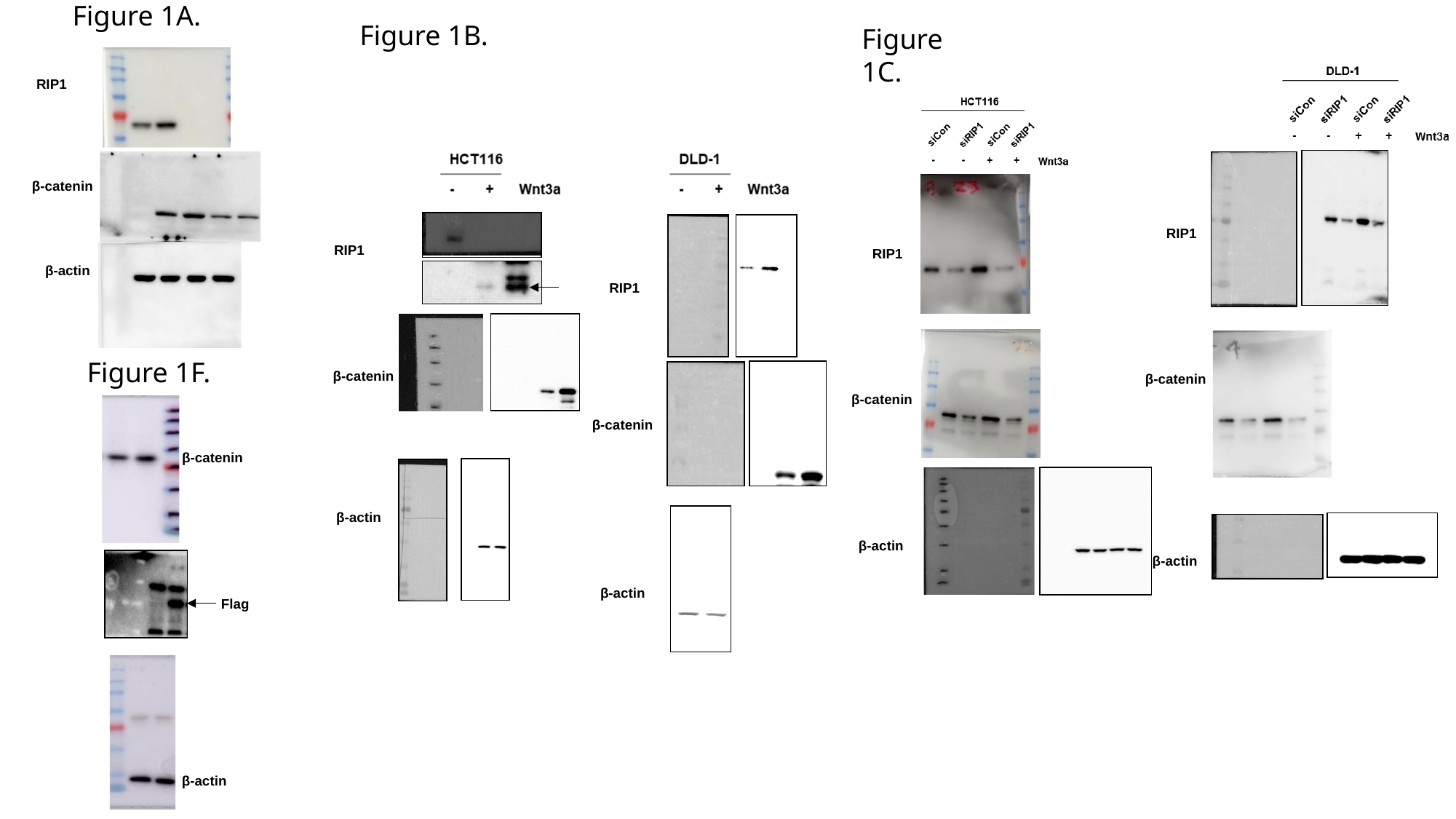

Figure 1A.
Figure 1B.
Figure 1C.
RIP1
β-catenin
RIP1
RIP1
RIP1
β-actin
RIP1
Figure 1F.
β-catenin
β-catenin
β-catenin
β-catenin
β-catenin
β-actin
β-actin
β-actin
β-actin
Flag
β-actin

## Slide 2
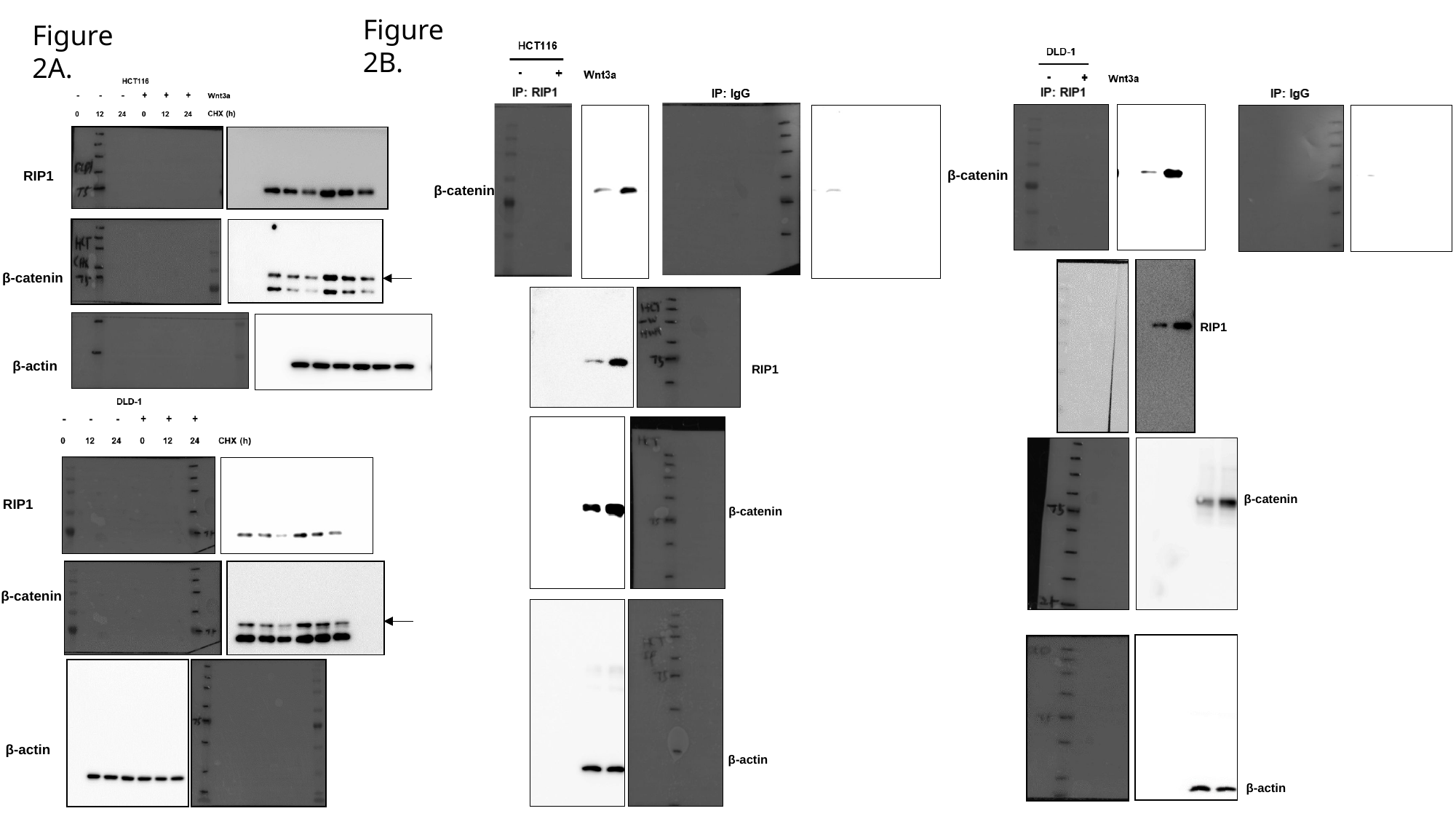

Figure 2B.
Figure 2A.
β-catenin
RIP1
β-catenin
β-catenin
RIP1
β-actin
RIP1
β-catenin
RIP1
β-catenin
β-catenin
β-actin
β-actin
β-actin

## Slide 3
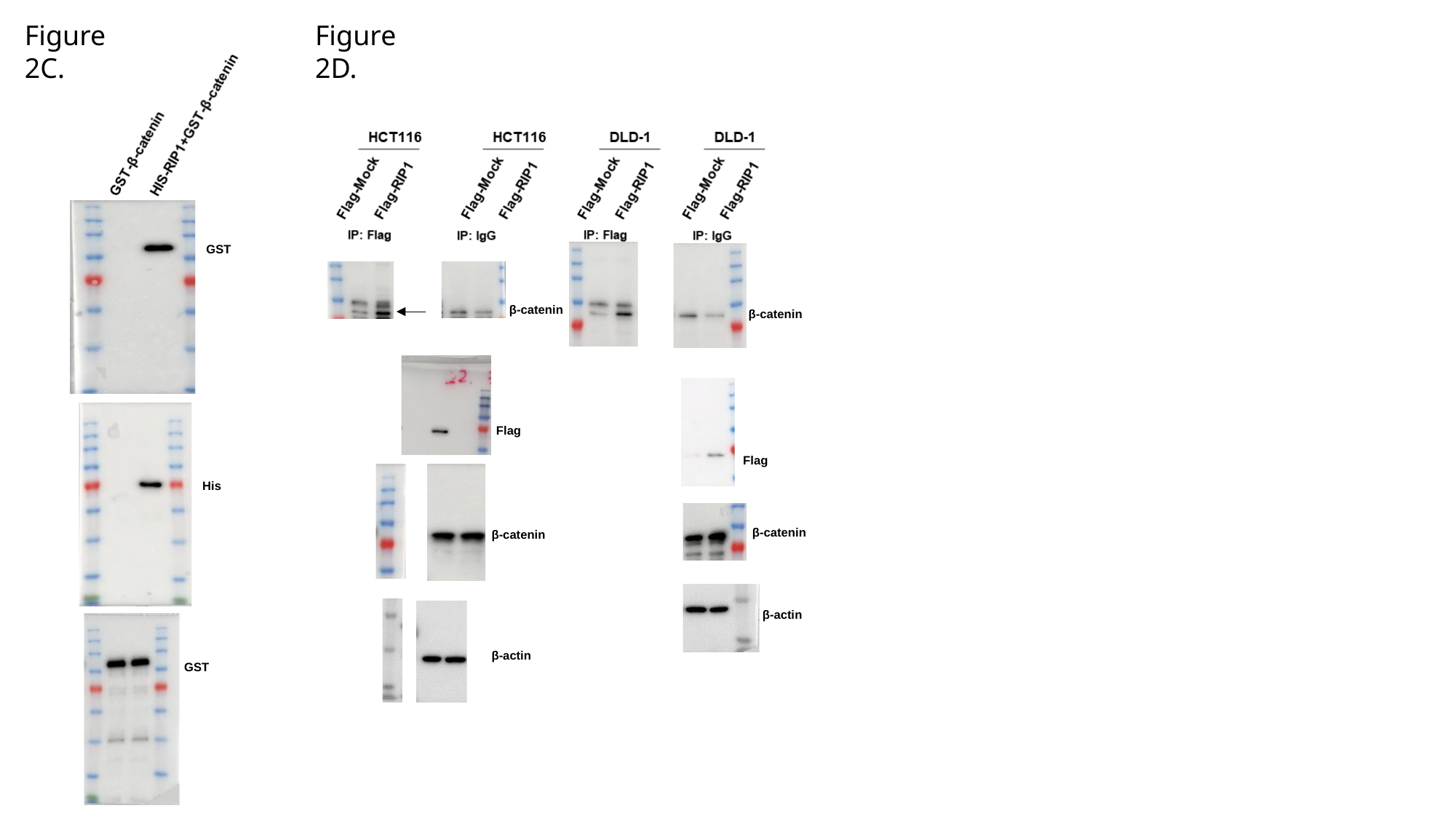

Figure 2C.
Figure 2D.
GST
β-catenin
β-catenin
Flag
Flag
His
β-catenin
β-catenin
β-actin
β-actin
GST

## Slide 4
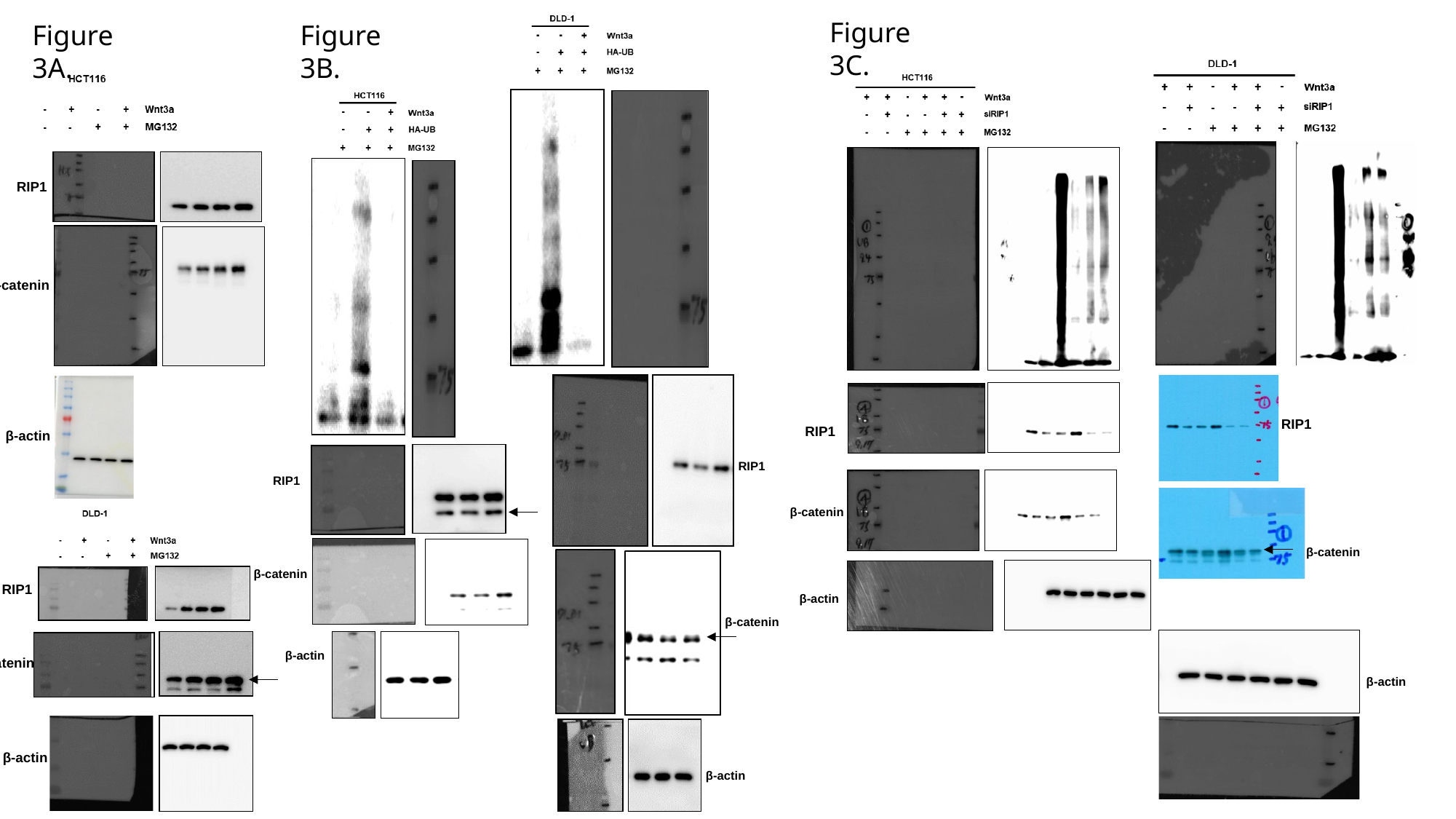

Figure 3C.
Figure 3A.
Figure 3B.
RIP1
β-catenin
RIP1
RIP1
β-actin
RIP1
RIP1
β-catenin
β-catenin
β-catenin
RIP1
β-actin
β-catenin
β-actin
β-catenin
β-actin
β-actin
β-actin

## Slide 5
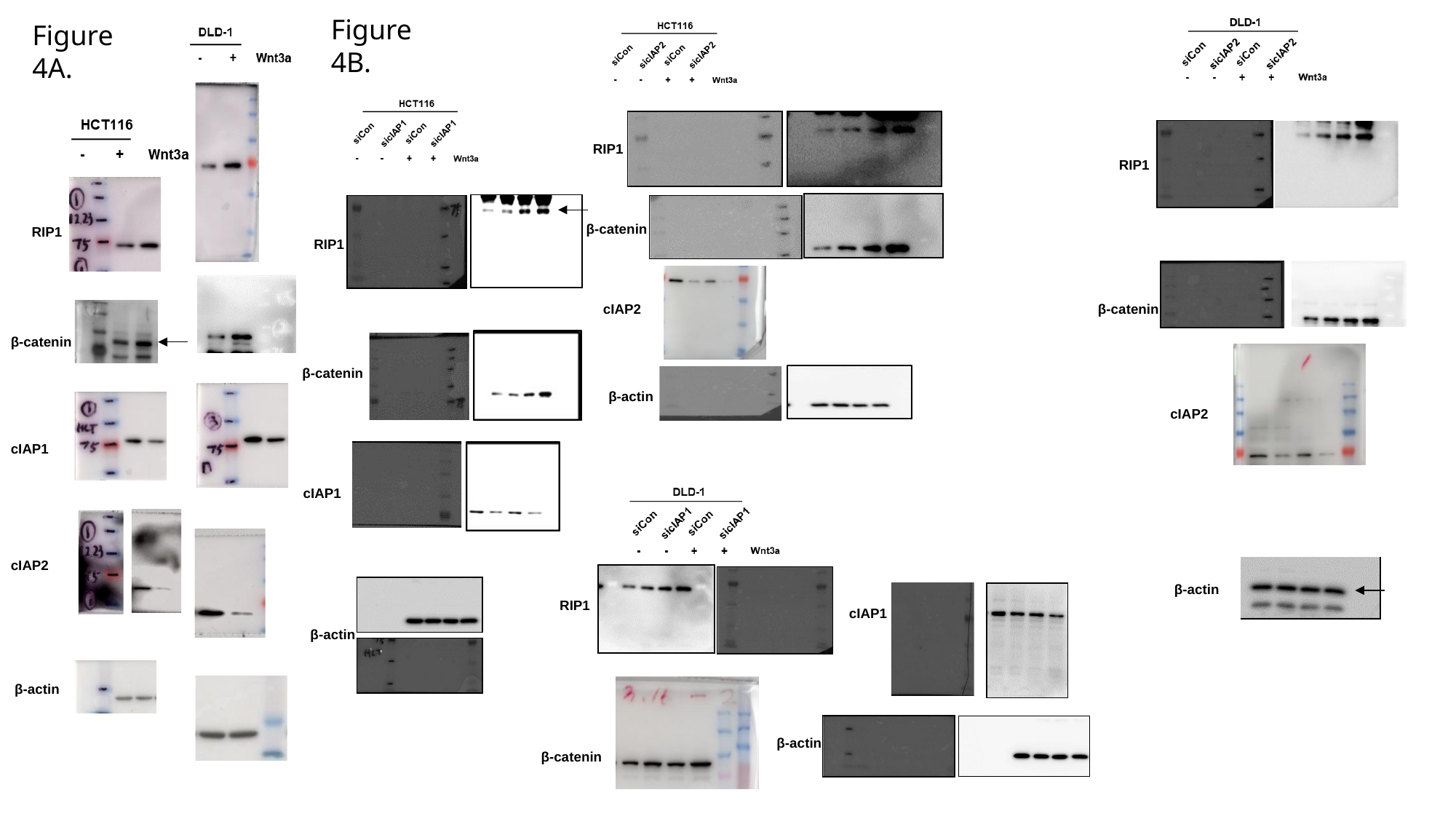

Figure 4B.
Figure 4A.
RIP1
RIP1
β-catenin
RIP1
RIP1
β-catenin
cIAP2
β-catenin
β-catenin
β-actin
cIAP2
cIAP1
cIAP1
cIAP2
β-actin
RIP1
cIAP1
β-actin
β-actin
β-actin
β-catenin

## Slide 6
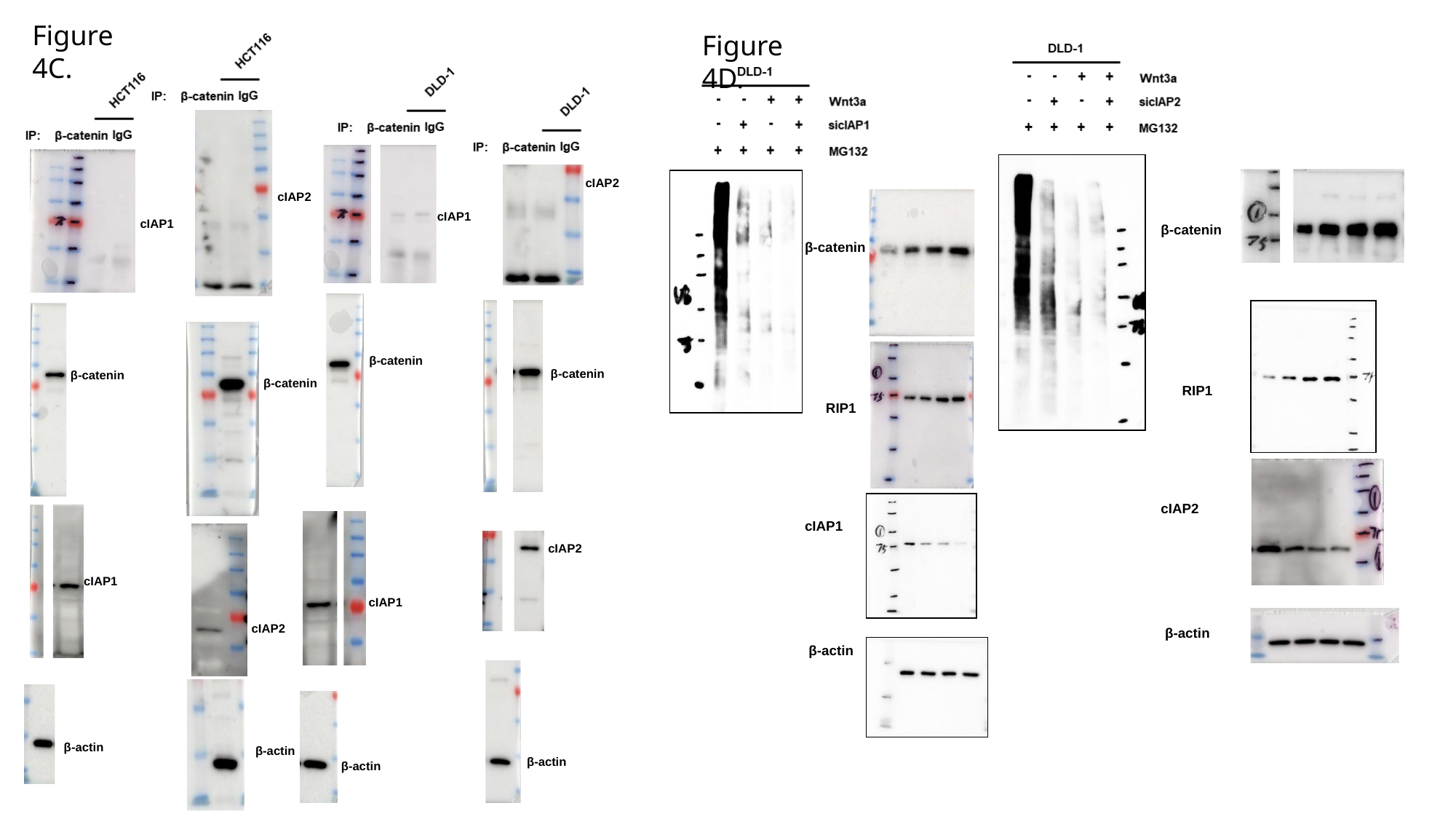

Figure 4C.
Figure 4D.
cIAP2
cIAP2
cIAP1
cIAP1
β-catenin
β-catenin
β-catenin
β-catenin
β-catenin
β-catenin
RIP1
RIP1
cIAP2
cIAP1
cIAP2
cIAP1
cIAP1
cIAP2
β-actin
β-actin
β-actin
β-actin
β-actin
β-actin

## Slide 7
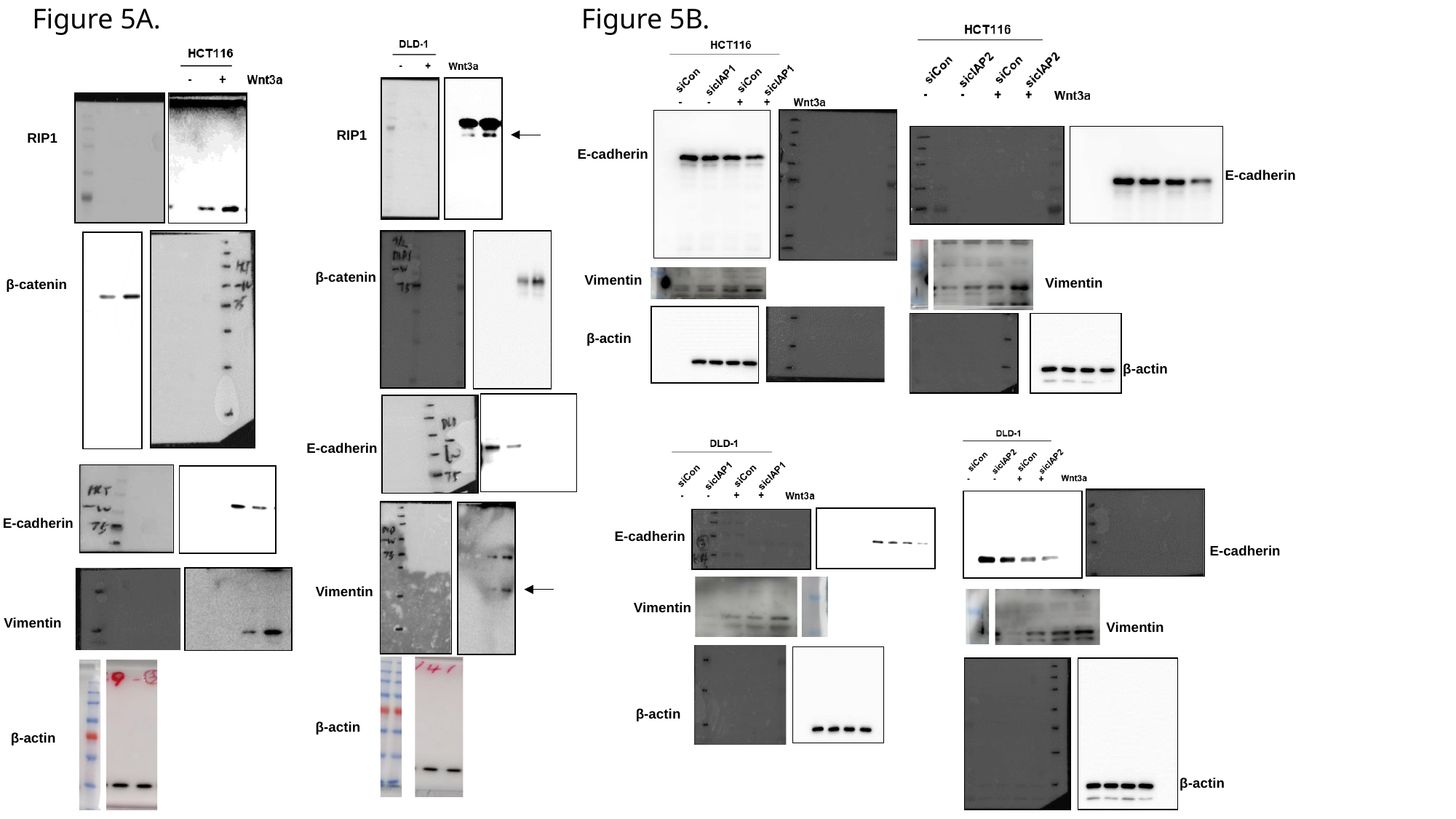

Figure 5A.
Figure 5B.
RIP1
RIP1
E-cadherin
E-cadherin
β-catenin
Vimentin
Vimentin
β-catenin
β-actin
β-actin
E-cadherin
E-cadherin
E-cadherin
E-cadherin
Vimentin
Vimentin
Vimentin
Vimentin
β-actin
β-actin
β-actin
β-actin

## Slide 8
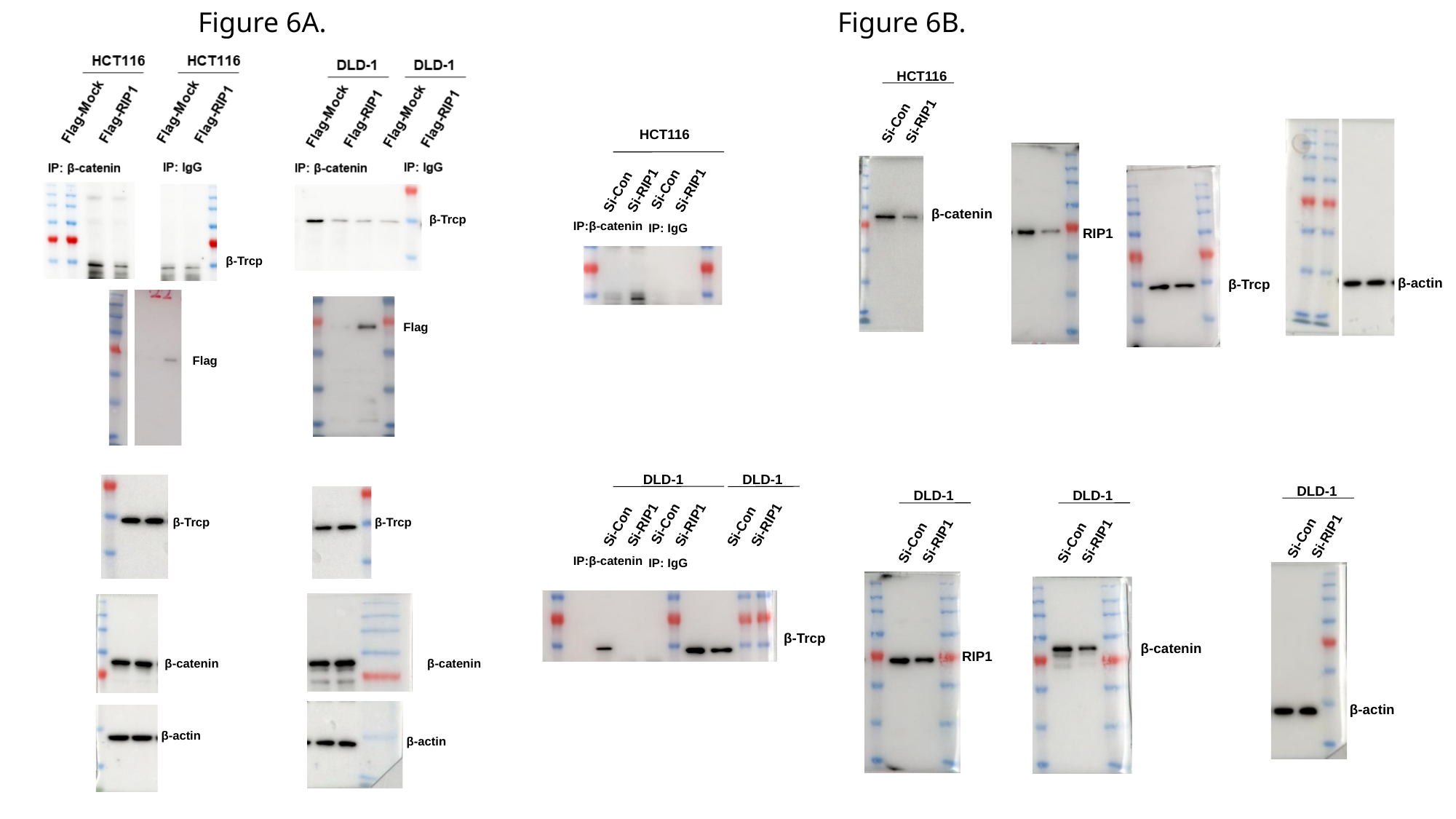

Figure 6A.
Figure 6B.
HCT116
Si-RIP1
Si-Con
HCT116
Si-Con
Si-RIP1
Si-RIP1
Si-Con
β-catenin
β-Trcp
IP:β-catenin
IP: IgG
RIP1
β-Trcp
β-actin
β-Trcp
Flag
Flag
DLD-1
DLD-1
DLD-1
DLD-1
DLD-1
Si-Con
Si-RIP1
Si-RIP1
Si-RIP1
Si-Con
Si-Con
Si-RIP1
β-Trcp
β-Trcp
Si-Con
Si-RIP1
Si-RIP1
Si-Con
Si-Con
IP:β-catenin
IP: IgG
β-Trcp
β-catenin
RIP1
β-catenin
β-catenin
β-actin
β-actin
β-actin

## Slide 9
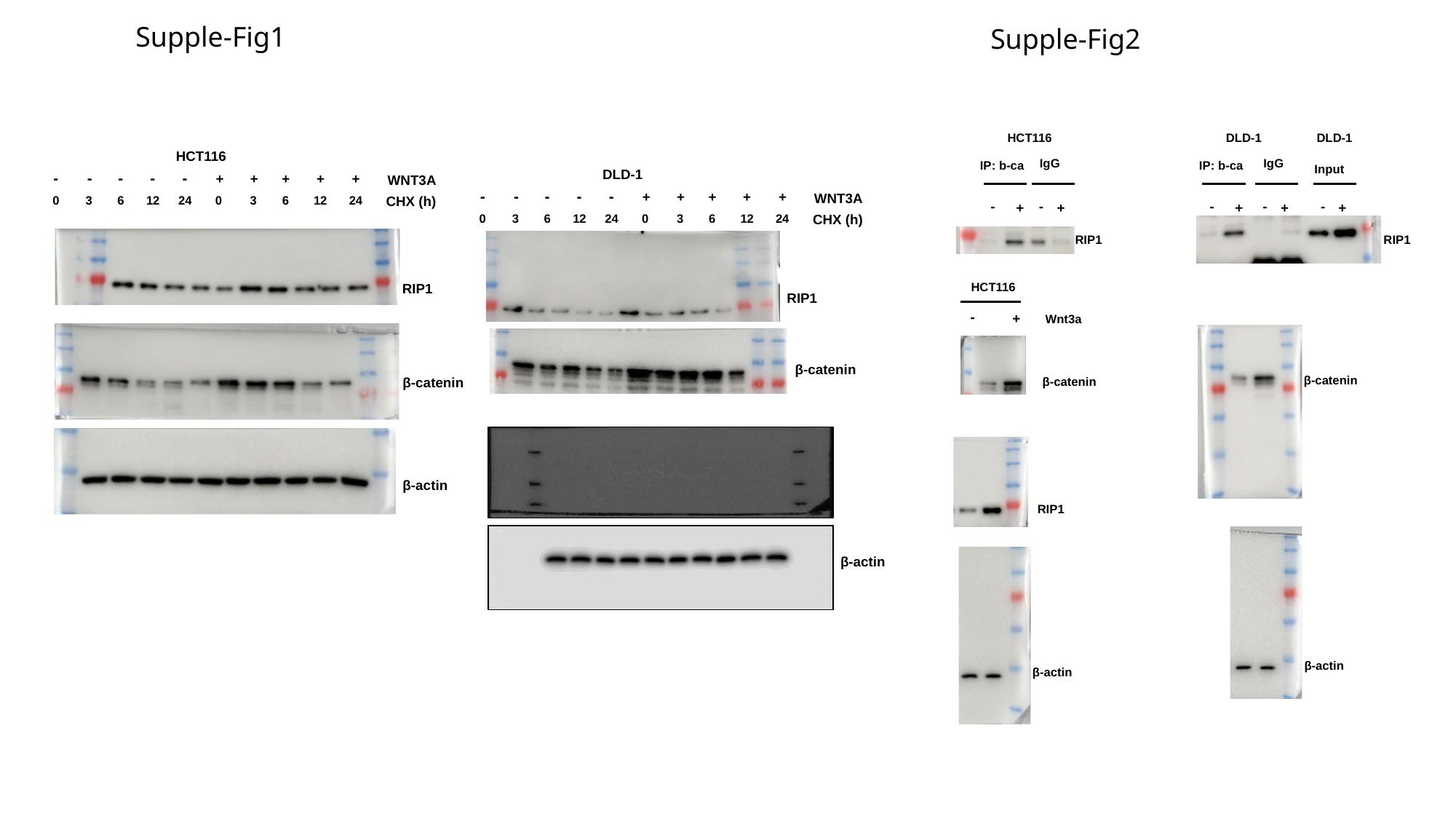

Supple-Fig1
Supple-Fig2
HCT116
DLD-1
DLD-1
HCT116
IgG
IgG
IP: b-ca
IP: b-ca
Input
DLD-1
 -
 -
 -
 -
 -
 +
 +
 +
 +
 +
WNT3A
 -
 -
 -
 -
 -
 +
 +
 +
 +
 +
WNT3A
0
3
6
12
24
0
3
6
12
24
CHX (h)
-
-
-
-
-
+
+
+
+
+
0
3
6
12
24
0
3
6
12
24
CHX (h)
RIP1
RIP1
HCT116
RIP1
RIP1
-
+
Wnt3a
β-catenin
β-catenin
β-catenin
β-catenin
β-actin
RIP1
β-actin
β-actin
β-actin
